# Temperature-Dependent Kinetics of the Reactions of the Criegee Intermediate CH_2_OO with Hydroxyketones

**DOI:** 10.1021/acs.jpca.4c00156

**Published:** 2024-03-01

**Authors:** Zachary A. Cornwell, Jonas J. Enders, Aaron W. Harrison, Craig Murray

**Affiliations:** † Department of Chemistry, University of California, Irvine, Irvine, California 92697, United States; ‡ Department of Chemistry, Austin College, Sherman, Texas 75090, United States

## Abstract

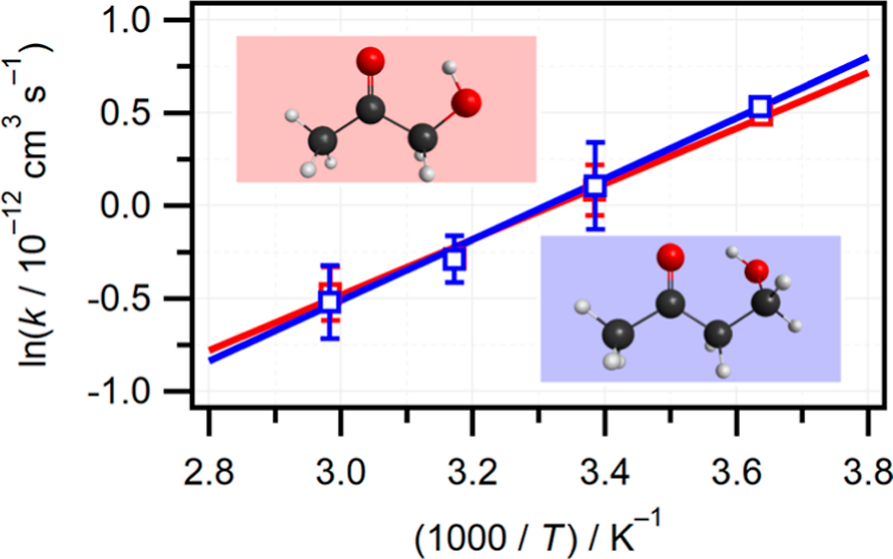

Though there is a growing body of literature on the kinetics of CIs with simple carbonyls, CI reactions with functionalized carbonyls such as hydroxyketones remain unexplored. In this work, the temperature-dependent kinetics of the reactions of CH_2_OO with two hydroxyketones, hydroxyacetone (AcOH) and 4-hydroxy-2-butanone (4H2B), have been studied using a laser flash photolysis transient absorption spectroscopy technique and complementary quantum chemistry calculations. Bimolecular rate constants were determined from CH_2_OO loss rates observed under pseudo-first-order conditions across the temperature range 275–335 K. Arrhenius plots were linear and yielded *T*-dependent bimolecular rate constants: *k*_AcOH_(*T*) = (4.3 ± 1.7) × 10^–15^ exp[(1630 ± 120)/*T*] and *k*_4H2B_(*T*) = (3.5 ± 2.6) × 10^–15^ exp[(1700 ± 200)/*T*]. Both reactions show negative temperature dependences and overall very similar rate constants. Stationary points on the reaction energy surfaces were characterized using the composite CBS-QB3 method. Transition states were identified for both 1,3-dipolar cycloaddition reactions across the carbonyl and 1,2-insertion/addition at the hydroxyl group. The free-energy barriers for the latter reaction pathways are higher by ∼4–5 kcal mol^–1^, and their contributions are presumed to be negligible for both AcOH and 4H2B. The cycloaddition reactions are highly exothermic and form cyclic secondary ozonides that are the typical primary products of Criegee intermediate reactions with carbonyl compounds. The reactivity of the hydroxyketones toward CH_2_OO appears to be similar to that of acetaldehyde, which can be rationalized by consideration of the energies of the frontier molecular orbitals involved in the cycloaddition. The CH_2_OO + hydroxyketone reactions are likely too slow to be of significance in the atmosphere, except at very low temperatures.

## Introduction

Criegee intermediates (CIs) are a zwitterionic species formed in alkene ozonolysis that can impact the oxidizing capacity of the troposphere.^1−4^ Alkene ozonolysis proceeds via a 1,3-dipolar cycloaddition reaction to produce a cyclic 1,2,3-trioxolane, or primary ozonide (POZ), that promptly decomposes to form a CI and a carbonyl compound.^1,5^ While larger CIs tend to undergo unimolecular decomposition,^6,7^ generating OH radicals, the smallest CI, CH_2_OO, has a longer lifetime and may undergo bimolecular reaction with trace atmospheric gases after collisional stabilization.^8,9^ Reaction with water vapor, primarily in the form of water dimer, is the major reactive sink for CH_2_OO,^4^ although bimolecular reactions with other trace atmospheric gases, such as SO_2_ and organic acids, are occasionally competitive in specific environments and under favorable conditions.^4,10^

CIs react with carbonyl species in a concerted 1,3-dipolar cycloaddition reaction to form a cyclic 1,2,4-trioxolane, or secondary ozonide (SOZ). Recently, work in our laboratory has explored the effect of varying the carbonyl substituents (R_1_R_2_CO, where R_1_ and R_2_ are alkyl or acyl groups) on the gas-phase reactivity of a series of ketones and diketones with CH_2_OO.^11,12^ Reactivity trends can be rationalized using concepts from frontier molecular orbital (FMO) theory.^13−16^ The cycloaddition mechanism arises primarily from π–π* interactions between the occupied *n*(*p*_C_–*p*_O_) orbital of the electron-rich species (CH_2_OO) and the unoccupied π* orbital of the electron-deficient species (R_1_R_2_CO). Electron-withdrawing groups (EWGs) on the carbonyl lower the energy of the carbonyl π* orbital, which reduces the energy gap with the CI orbital, stabilizes the transition state (TS), and increases the reactivity. Electron-donating groups (EDGs) have the opposite effect and ultimately decrease reactivity. Hammett substituent constants provide a useful qualitative proxy for the electron-donating or withdrawing-character of the substituents on the carbonyl.^17,18^

The kinetics of the reaction of acetone (Ac, R_1_ = R_2_ = CH_3_), a representative model ketone, with CH_2_OO has been thoroughly investigated experimentally,^11,12,19−22^ with recent measurements converging on a 298 K rate constant of ∼5 × 10^–13^ cm^3^ s^–1^. Aldehydes (R_1_ = H, R_2_ = H or alkyl) react faster, with rate constants in the range (1–4) × 10^–12^ cm^3^ s ^–1^.^19,20,23−25^ The presence of strongly EWGs increases rate constants further. For example, hexafluoroacetone (HFA, R_1_ = R_2_ = CF_3_) has a rate constant of ∼3 × 10^–11^ cm^3^ s^–1^.^19,26^ The α-diketones, biacetyl (BiAc, R_1_ = CH_3_, R_2_ = CH_3_CO), and acetyl propionyl (AcPr, R_1_ = CH_3_/C_2_H_5_, R_2_ = C_2_H_5_CO/CH_3_CO) have both electron-donating alkyl and electron-withdrawing acyl substituents, and the reactions with CH_2_OO have rate constants of ∼1 × 10^–11^ cm^3^ s^–1^.^11,12^ Where examined, all reactions of carbonyls with CH_2_OO show a negative temperature dependence.

Our focus in this study is the reactions of CH_2_OO with two hydroxyketones: hydroxyacetone (acetol, AcOH, R_1_ = CH_3_, R_2_ = CH_2_OH) and 4-hydroxy-2-butanone (4H2B, R_1_ = CH_3_, R_2_ = CH_2_CH_2_OH). Hydroxyketones are multifunctional VOCs, which present multiple reactive sites and may exhibit cooperative effects.^27^ The reactions of AcOH and 4H2B with CH_2_OO can occur at the carbonyl or the hydroxyl moieties. The latter pathway is expected to be minor, as aliphatic alcohols react with CH_2_OO to form alkoxymethyl hydroperoxides, with rate constants in the range (1–2) × 10^–13^ cm^3^ s^–1^ at 298 K,^28,29^ smaller than those of most carbonyls. The hydroxymethyl (CH_2_OH) group has Hammett substituent constants of zero and is neither electron-donating nor withdrawing. Consequently, the rate constant for the cycloaddition reaction is anticipated to be comparable to that of acetaldehyde.

AcOH and 4H2B have also been identified as trace gases in the troposphere,^30,31^ where they are formed as secondary oxidation products of isoprene and other atmospheric hydrocarbons.^32−35^ AcOH is also produced directly from biomass burning.^36,37^ The major reactive sink for hydroxyketones is reaction with OH radicals, which results in lifetimes of a few days. Kinetics studies of the OH + AcOH reaction have produced surprisingly inconsistent results; the IUPAC recommendation for the 298 K rate constant is 5.9 × 10^–12^ cm^3^ s^–1^.^38−47^ The OH + 4H2B kinetics measurements show similar inconsistencies, but the 298 K rate constant appears to be similar.^40,48−52^ Photolysis of both species is far slower than reaction with OH, particularly for AcOH where the presence of the α-hydroxyl group causes a ∼10 nm blue-shift of the first absorption band that reduces the absorption at actinic wavelengths (see Figure S2 in the Supporting Information).^53^

The results of laser flash photolysis transient absorption spectroscopy measurements quantifying the temperature-dependent kinetics of the reactions of CH_2_OO with AcOH and 4H2B across the range 275–335 K are reported here. Complementary ab initio calculations map out the reaction energy profiles and provide a basis for explaining reactivity trends using FMO theory. The potential implications of the title reactions in the atmosphere are briefly discussed.

## Methods

The temperature-controlled flash photolysis, transient absorption spectroscopy apparatus has been described in detail previously^12^ and will be summarized briefly here.

CH_2_OO was produced in the flow reactor by the photolysis of diiodomethane (CH_2_I_2_) in the presence of excess O_2_ using the 355 nm output of an Nd:YAG laser (Continuum Surelite II-10). Typical pulse energies were ∼10 mJ, resulting in fluences of ∼28 mJ cm^–2^. Absorption spectra were obtained by dispersing the output of pulsed LEDs (LightSpeed Technologies) in a spectrograph (Andor Shamrock 303i with iDus 420 CCD camera). Kinetics measurements used an LED nominally centered at 365 nm to obtain transient absorption spectra of CH_2_OO (and IO) in the range 360–395 nm at various time delays after photolysis. Independent absorption measurements were performed in the range 270–295 nm using an LED centered at 280 nm to quantify reactant concentrations. A digital delay generator (Quantum Composers, 9528) synchronized the photolysis laser, LED driver, and CCD camera.

The flow reactor itself comprises a jacketed quartz tube, with an effective path length of 90 cm. A unistat (Huber Tango) precisely controlled the reactor temperature (within <1 K) over the range 275–335 K. Gas flows into the reactor were controlled using a range of choked-flow orifices (O’Keefe). Gases (O_2_ and N_2_) were used directly from the cylinders, while liquids (CH_2_I_2_, AcOH, and 4H2B) were placed in smog bubblers and carried into the cell by a flow of N_2._ The smog bubblers were held in a water bath maintained at 295 K to prevent evaporative cooling and vapor pressure drop off. Measurements with AcOH used a total flow rate of 3.8 sLpm, resulting in a pressure of 78 Torr in the reactor. The lower vapor pressure of 4H2B (1.2 Torr versus 3.5 Torr at 295 K)^49,54^ required a larger total flow rate of 4.9 sLpm and a reactor pressure of 100 Torr. Typically, the concentrations in the reactor were [CH_2_I_2_] = 1.1 × 10^15^ cm^–3^, [O_2_] = 2.1 × 10^17^ cm^–3^, [AcOH] = (1–5) × 10^15^ cm^–3^, and [4H2B] = (0.5–1.5) × 10^15^ cm^–3^, with N_2_ balance. All chemicals were used as supplied: O_2_ (Airgas, UHP 4.4), N_2_ (Airgas, industrial grade), CH_2_I_2_ (Sigma-Aldrich, 99%), AcOH (Acros Organics, 90%), and 4H2B (Tokyo Chemical Industry, 95%). FT-IR spectra of the headspace above samples of liquid AcOH and 4H2B were recorded (see Figure S1 in Supporting Information) using a JASCO 4700 spectrometer to identify any impurities. No bands associated with any other organic species were identified, consistent with previous suggestions that the likely impurity in AcOH is residual H_2_O,^55^ although at levels too low to affect the kinetics measurements.

Electronic structure calculations were performed with the GAMESS and Gaussian 16 programs.^56−59^ Geometries of reactants, products, entrance channel complexes, and TS structures were initially optimized using the B3LYP functional with the Dunning-type cc-pVDZ basis set and the harmonic frequencies subsequently calculated. The presence of zero or one imaginary frequency confirmed that the optimized geometries were true minima or TSs, respectively. Intrinsic reaction coordinate (IRC) calculations were performed to verify that the expected reactants and products were reached on either side of the TS. Reaction thermochemistry was determined using rigid-rotor harmonic-oscillator (RRHO) partition functions. Additional calculations were performed using the composite CBS-QB3 method to refine the calculated energies.^60^ The CBS-QB3 method provides reliable thermochemistry at modest computational cost.^61−63^ In addition, the FMO energies are obtained from the optimization output at the B3LYP/cc-pVDZ level of theory. Previous work has shown the frontier orbital energies calculated at a similar level of theory to provide linear correlation with molecular properties such as ionization potential, electron affinity, and excitation energy.^64^

## Results

### Reactant Concentration Measurements

As in our previous work,^11,12,28^ concentrations of the hydroxyketone reactants in the flow cell during kinetics measurements can be estimated using reported vapor pressures (3.50 ± 0.17 Torr for AcOH, and 1.24 ± 0.04 Torr for 4H2B)^49,54^ and fractional flow rates. Since the first UV absorption bands of both hydroxyketones can be observed using an LED centered at 280 nm,^53^ their concentrations can also be measured directly. Absorption spectra of the hydroxyketones are recorded in the wavelength range 275–290 nm under conditions that are otherwise identical to those used in the kinetics measurements across the 275–335 K temperature range. Absolute hydroxyketone number densities are determined using previously reported absorption cross sections. The first UV absorption bands of AcOH and 4H2B have peak cross sections of ∼6 × 10^–20^ cm^2^ at ∼270 and 280 nm, respectively, as is typical of the excitation to the S_1_(nπ*) state in carbonyls.^53^ The only reported measurement for 4H2B is by Messaadia et al.,^65^ while various measurements exist for AcOH.^39,42,53,65^ There is a discrepancy between the AcOH cross sections recommended by the IUPAC Task Group on Atmospheric Chemical Kinetic Data Evaluation and JPL Chemical Kinetics and Photochemical Data for Use in Atmospheric Studies Evaluation.^47,66^ While IUPAC prefers the values of Orlando et al.,^39^ JPL uses an average of that and lower values measured by Butkovskaya et al.^39,42^ Since the other reported measurements agree well with the JPL recommendation,^53,65^ we elect to use it to quantify [AcOH]_exp_ and determine a concentration scale factor. The UV absorption spectra of both hydroxyketones are shown in Figure S2 in the Supporting Information.

**Figure 1:**
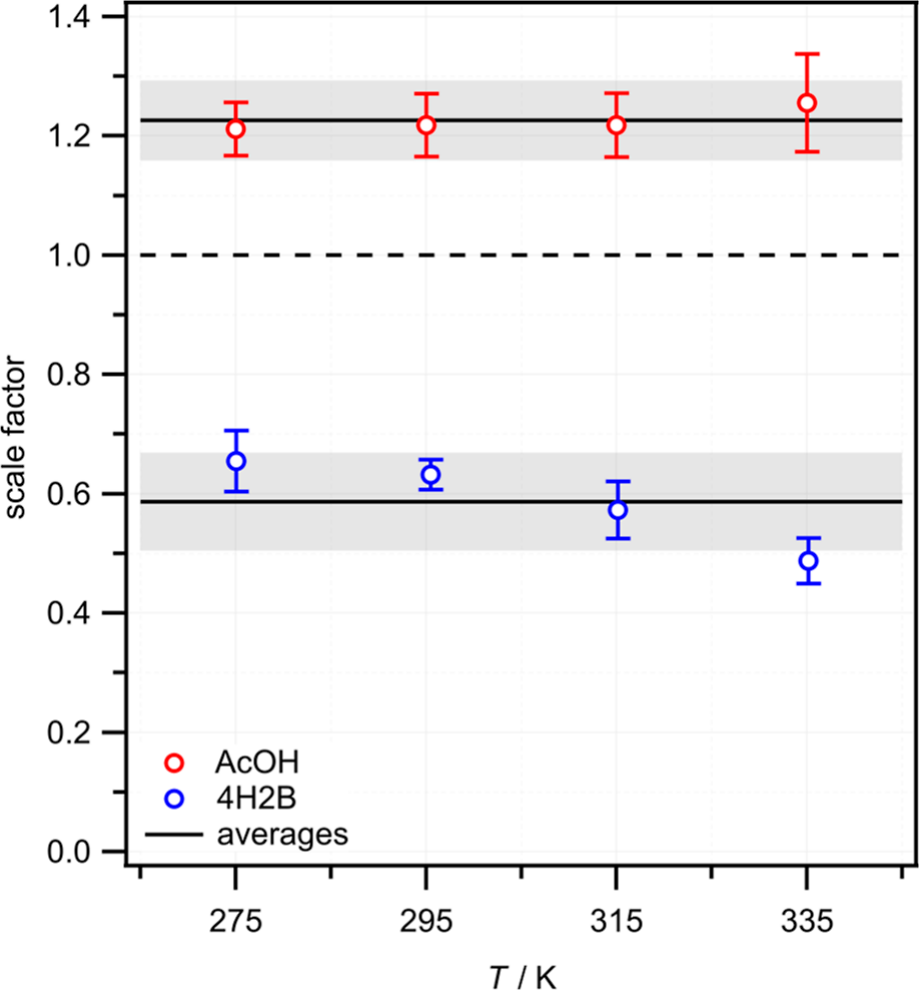
Scale factors determined from gradient of [X]_exp_ versus [X]_est_ calibration plots as a function of temperature. Solid lines are the *T*-independent average, while shaded areas represent the estimated experimental uncertainty, based on the variability of the measurements. Values >1 or <1 indicate that reactant concentration is underestimated (AcOH) or overestimated (4H2B), respectively.

The gradients of plots of measured against estimated concentrations ([X]_exp_ vs [X]_est_) (Figures S3 and S4 in the Supporting Information) provide a scaling factor that can be used to correct the estimated hydroxyketone concentrations. Previously,^11,12^ we have found concentration scaling factor values within 10% of unity for acetone and diketones, indicating that the estimates give values close to the actual concentrations in the flow cell. The hydroxyketone concentration scaling factors deviate from unity, however, as can be seen from Figure 1. For AcOH, the scale factor is independent of temperature with an average value of 1.23 ± 0.07, indicating that the actual concentration is slightly higher than estimated. In contrast, the values determined for 4H2B suggest that its concentration is overestimated, with an average of 0.59 ± 0.08. The overall uncertainties are estimated from the spread in values obtained in multiple measurements across the temperature range.

Deviations from unity for the concentration scaling factors derive primarily from systematic errors in [X]_est_ and/or [X]_exp_, which depend respectively on the hydroxyketone vapor pressures *P*_vap,X_ and absorption cross sections σ_X_(λ). The *T*-dependent vapor pressures of AcOH and 4H2B have been measured to a high degree of precision,^49,54^ although several groups have discussed evidence of hydroxyketone “stickiness” in the course of kinetics measurements.^43,44,65^ Wall losses between the bubbler and the flow reactor would lead to concentration overestimates and scaling factors <1, which is consistent with the observed value for 4H2B but not for AcOH. Based on the FT-IR spectrum of AcOH shown in Figure S1 in the Supporting Information and reported band intensities,^36^ we estimate a vapor pressure of 3.9 ± 0.4 Torr. If the C=O stretch bands of AcOH and 4H2B are assumed to have the same intensity (as supported by ab initio calculations), we estimate the vapor pressure of 4H2B to be 0.76 ± 0.11 Torr. The ratios of these estimated vapor pressures to the literature values^49,54^ are 1.11 ± 0.12 for AcOH and 0.61 ± 0.09, in good agreement with the measured scaling factors and suggesting that the systematic error originates in the reported values of *P*_vap,X_. Another possibility for values <1 is that the smog bubbler headspace is not saturated with the organic vapor, although the use of the experimental scale factor corrects for this effect.

For the purposes of the kinetics measurements, the absorption cross sections are ultimately of most significance as they are used to determine [X]_exp_ directly. As noted above, σ_AcOH_(λ) values recommended by IUPAC are 10% greater than the JPL values, which would produce lower values of [AcOH]_exp_ and bring the concentration scaling factor value closer to unity (1.12 ± 0.06) and closer to the vapor pressure ratio estimated from the FT-IR spectra. Such a change would also require an increase in the bimolecular rate constant for reaction with CH_2_OO proportionally. We proceed on the basis that the σ_X_(λ) values used are accurate. The rate constants determined in the experiments discussed below are inversely proportional to σ_X_(λ) and can be adjusted appropriately if improved values become available in the future.

### Kinetics Measurements

The kinetics of the reactions of CH_2_OO with AcOH and 4H2B were studied under pseudo-first-order conditions of excess hydroxyketone at four temperatures in the range 275–335 K. The lowest hydroxyketone concentrations were approximately 2 orders of magnitude greater than [CH_2_OO]_0_. Transient absorption spectra obtained in the range 363–395 nm at various photolysis-probe delay times were decomposed into contributions from CH_2_OO and IO using known absorption spectra^67,68^ to generate [CH_2_OO]_*t*_ and [IO]_*t*_ concentration–time profiles. Examples of typical experimental transient spectra recorded with and without AcOH and the resulting [CH_2_OO]_*t*_ profiles are shown in Figure S5 in the Supporting Information. As expected, the CH_2_OO concentrations are observed to decrease more rapidly with increasing hydroxyketone concentration, while the IO concentration profiles remain unaffected. Peak CI concentrations of [CH_2_OO]_0_ = (6–8) × 10^12^ cm^–3^ are significantly smaller than those of the hydroxyketone reactants, ensuring pseudo-first-order conditions.

**Figure 2:**
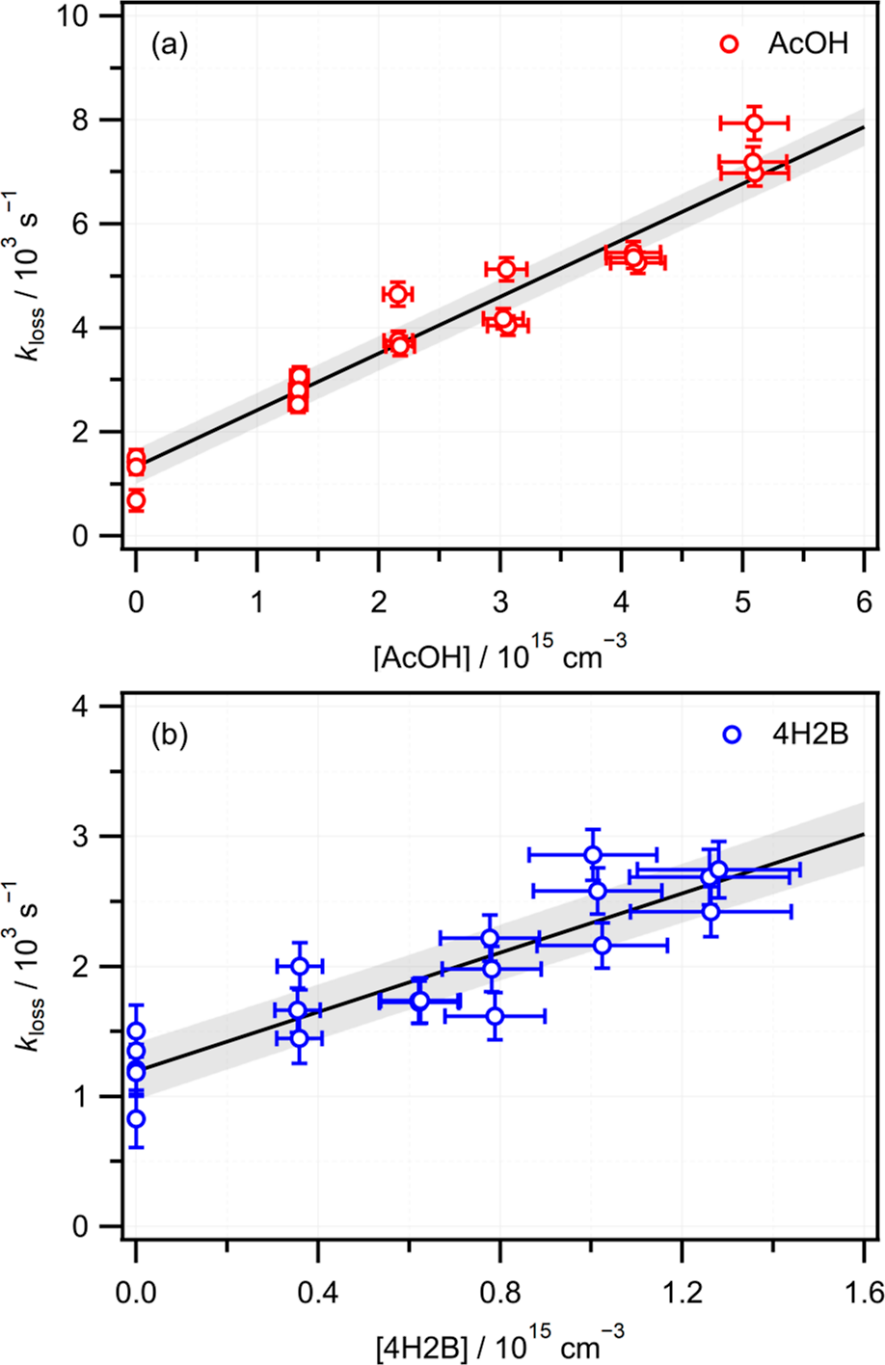
Pseudo-first-order plots for the reactions of CH_2_OO with (a) AcOH and (b) 4H2B at 295 K. Vertical and horizontal error bars represent statistical uncertainties (1σ) in the loss rates determined from fitting [CH_2_OO] time profiles and uncertainties in the hydroxyketone concentration calibration measurements, respectively. Weighted linear fits to the experimental data are also shown, with shaded area representing 1σ prediction bands.

Analysis of the [CH_2_OO]_*t*_ profiles used the same kinetic model as described previously and summarized in Supporting Information.^11,12^ The differential rate law for CH_2_OO loss includes a quadratic term for bimolecular self-reaction with rate constant *k*_self_ and a linear term for pseudo-first-order reactions with rate constant *k*_loss_. The pseudo-first-order rate constants *k*_loss_ at each hydroxyketone concentration are derived from fits of the [CH_2_OO]_*t*_ profiles to the integrated rate law, with *k*_self_ fixed to a *T*-independent value of 7.8 × 10^–11^ cm^3^ s^–1^.^12^ Plots of *k*_loss_ against [hydroxyketone] are linear, and a least-squares fit (weighted by the uncertainties in both *k*_loss_ and [hydroxyketone]) returns a background loss rate *k*_bgd_ and the bimolecular rate constant *k*_hydroxyketone_ as the gradient. Examples are shown in Figure 2 for the reactions of CH_2_OO with AcOH and 4H2B at 295 K, and the full set of measurements at all four temperatures is shown in Figures S6 and S7 in the Supporting Information. At 295 K, the bimolecular rate constants for the CH_2_OO + AcOH and CH_2_OO + 4H2B reactions are the same within the statistical uncertainties of the fits: *k*_AcOH_ = (1.09 ± 0.15) × 10^–12^ cm^3^ s^–1^ and *k*_4H2B_ = (1.11 ± 0.26) × 10^–12^ cm^3^ s^–1^. As has been observed for other reactions of CH_2_OO with carbonyl species,^12,20,22,25^ the rate constants for the AcOH and 4H2B reactions also decrease with increasing temperature. The complete set of measured *T*-dependent rate constants is summarized in Table 1. Background loss rates, attributed to reaction with I atoms or other species present in the flow reactor, are typically between ∼1000 and 1500 s^–1^ and decrease slightly with increasing temperature. The bimolecular rate constants for the CH_2_OO + hydroxyketone reactions were obtained by averaging three individual kinetic runs at each temperature, with errors representing the statistical uncertainty (1σ) in the fit. The same values, although with smaller uncertainties, are obtained from global fits to the complete data sets at each temperature.

**Table 1:** *T*-dependent Bimolecular Rate Constants, Arrhenius Parameters, and Standard Enthalpies, Entropies, and Gibbs Energies of Activation at 298 K for the Reactions of CH_2_OO with Hydroxyacetone and 4H2B^a^

*T*/K	*k*_AcOH_/10^–^^12^ cm^3^ s^–^^1^	*k*_4H2B_/10^–^^12^ cm^3^ s^–^^1^
275	1.63 ± 0.07	1.70 ± 0.04
295	1.09 ± 0.15	1.11 ± 0.26
315	0.75 ± 0.03	0.75 ± 0.09
335	0.62 ± 0.09	0.60 ± 0.12
*A*/10^–^^15^ cm^3^ s^–^^1^	4.3 ± 1.7	3.5 ± 2.6
(*E*_a_/*R*)/K	–1630 ± 120	–1700 ± 200
*E*_a_/kcal mol^–^^1^	–3.24 ± 0.23	–3.38 ± 0.40
Δ^⧧^*H*°/kcal mol^–^^1^	–4.43 ± 0.23	–4.56 ± 0.40
Δ^⧧^*S*°/cal K^–^^1^ mol^–^^1^	–39.5 ± 0.4	–39.9 ± 0.7
Δ^⧧^*G*°/kcal mol^–^^1^	+7.36 ± 0.26	+7.34 ± 0.46

a Uncertainties are 1σ statistical uncertainties from the fits.

**Figure 3:**
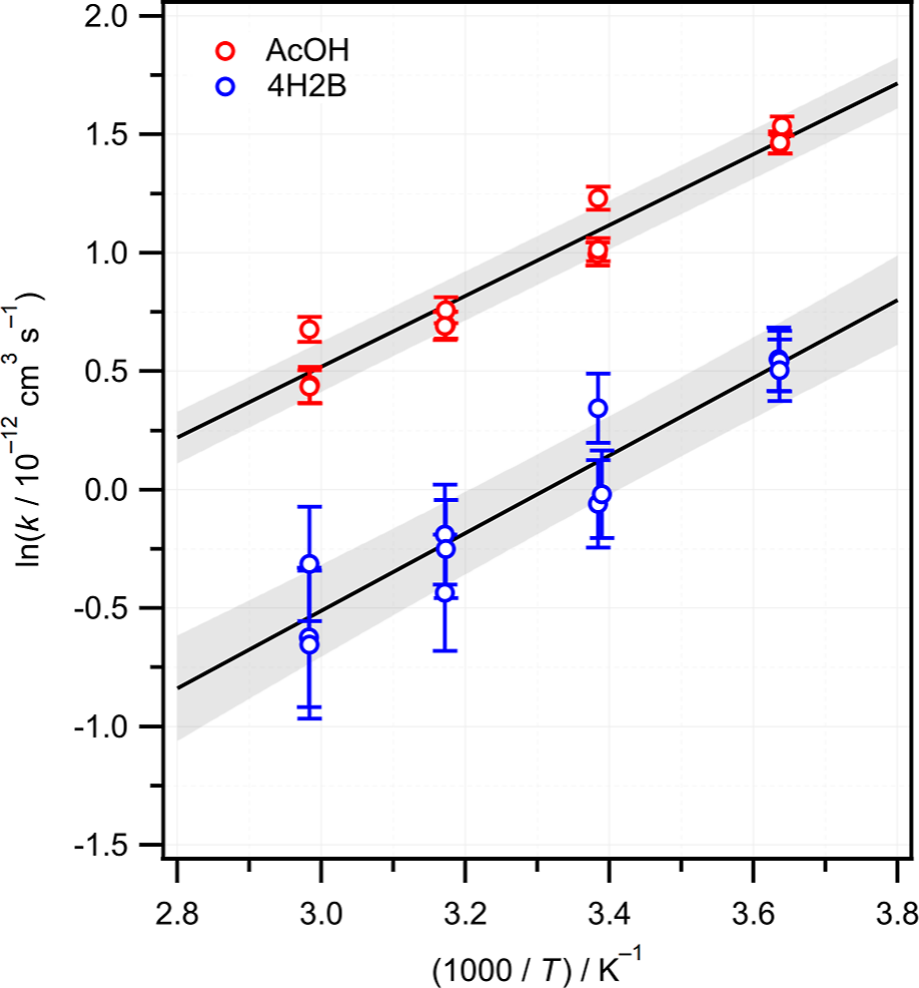
Arrhenius plots for the reaction of CH_2_OO with AcOH (red) and 4H2B (blue), with 1σ statistical uncertainties. The AcOH data have been offset vertically for clarity. Solid black lines are linear fits with shaded areas representing 1σ prediction bands.

Arrhenius plots for both reactions are shown in Figure 3. The plots are linear over the temperature range explored in the experiments, and the *T*-dependent bimolecular rate constants can be expressed in Arrhenius form as *k*_AcOH_(*T*) = (4.3 ± 1.7) × 10^–15^ exp[(1630 ± 120)/*T*] and *k*_4H2B_(*T*) = (3.5 ± 2.6) × 10^–15^ exp[(1700 ± 200)/*T*]. The similarity of the −*E*_a_/*R* values indicates that both reactions are equally sensitive to temperature, within the 1σ experimental uncertainties, with negative energies of activation of *E*_a_ ≈ −3.3 kcal mol^–1^.

The thermodynamic formulation of canonical transition state theory (CTST) for a bimolecular gas-phase reaction can be used to extract the standard entropy, enthalpy, and free energy of activation
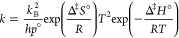


The factor *k*_B_^2^/*hp*° is evaluated to be 2.87 × 10^–12^ cm^3^ s^–1^ K^–2^ using *p*° = 10^5^ Pa. Δ^⧧^*S*° and Δ^⧧^*H*° are the standard entropy and enthalpy of activation, respectively. The latter is related to the activation energy by Δ^⧧^*H*° = *E*_*a*_ – 2*RT* for a bimolecular reaction.^69^ Unsurprisingly, given the similarities in the *T*-dependent rate constants, the thermodynamic parameters derived for the AcOH and 4H2B reactions are also similar, with Δ^⧧^*S*° ≈ −40 cal K^–1^ mol^–1^, Δ^⧧^*H*° ≈ −4.5 kcal mol^–1^, and Δ^⧧^*G*° ≈ +7.3 kcal mol^–1^ at 298 K. The pre-exponential factors, activation energies, and thermodynamics of activation for AcOH and 4H2B are compiled in Table 1. An alternative analysis using linear least-squares fits of ln (*k*/*T*^2^) vs 1/*T*, as shown in Figure S8 and Table S1 in Supporting Information, yields identical thermodynamic parameters within the experimental uncertainties.

The pressure dependence of the CH_2_OO + hydroxyketone reactions was also investigated by measuring *k*_loss_ across the 80–120 Torr range at 295 K and using a single concentration of each hydroxyketone ([AcOH] = (5.1 ± 0.3) × 10^15^ cm^–3^ and [4H2B] = (8.0 ± 1.1) × 10^14^ cm^–3^). The total pressure was changed by varying the flow rate of the N_2_ buffer gas, while leaving all other flow rates unchanged. The average *k*_loss_ values as a function of total pressure are shown in Figure S9 in Supporting Information. No change in *k*_loss_ is observed as the total pressure is varied, and the average values are the same as those obtained in the kinetics measurements. Based on similar measurements for the CH_2_OO + Ac reaction,^12,22^ we conclude that the rate constants measured in 80–100 Torr of N_2_ likely represent the high-pressure limit.

### Computational Results

We have performed ab initio calculations to characterize the reactions of CH_2_OO with AcOH and 4H2B. Geometries of the reactants, entrance channel complexes, TSs, and primary products were optimized, initially at the B3LYP/cc-pVDZ level of theory, and harmonic frequency analysis performed to confirm minima and saddle points. IRC calculations were performed to ensure that the TSs connected the reactant and product minima. Subsequent calculations were performed at the CBS-QB3 level of theory to provide improved thermochemistry. The Cartesian coordinates and energies for all species are compiled in the Supporting Information, and the CBS-QB3 thermochemistry data, Δ(*E* + ZPE) at 0 K and Δ*H*°, Δ*G*° at 298 K, are summarized in Table 2.

**Table 2:** CBS-QB3 Thermochemistry for the Reactions of CH_2_OO with AcOH and 4H2B: Δ*E* + ZPE @ 0 K (Δ*H*° @ 298 K) [Δ*G*° @ 298 K] in kcal mol^–1^

		cycloaddition A	cycloaddition B	1,2-addition
AcOH	vdW	–8.7 (−8.3) [+1.3]	–8.7 (−8.5) [+2.1]	–4.0 (−3.7) [+5.1]
	TS	–5.6 (−6.5) [+6.9]	–6.2 (−7.2) [+6.7]	+2.2 (+1.4) [+13.6]
	product	–48.1 (−49.5) [−34.9]	–48.3 (−49.8) [−34.8]	–48.0 (−48.9) [−35.9]
4H2B	vdW	–8.3 (−8.0) [+1.8]	–8.9 (−8.6) [+1.3]	–4.9(−4.6) [+4.1]
	TS	–5.7 (−6.5) [+6.4]	–6.7 (−7.6) [+5.6]	–0.5 (−1.1) [+10.3]
	product	–49.2 (−50.6) [−35.8]	–49.6 (−51.0) [−36.2]	–46.6 (−46.9) [−36.1]
Ac	TS	–6.1 (−6.4) [+5.5]		
MeOH	TS			–2.0 (−3.1) [+9.1]

**Figure 4:**
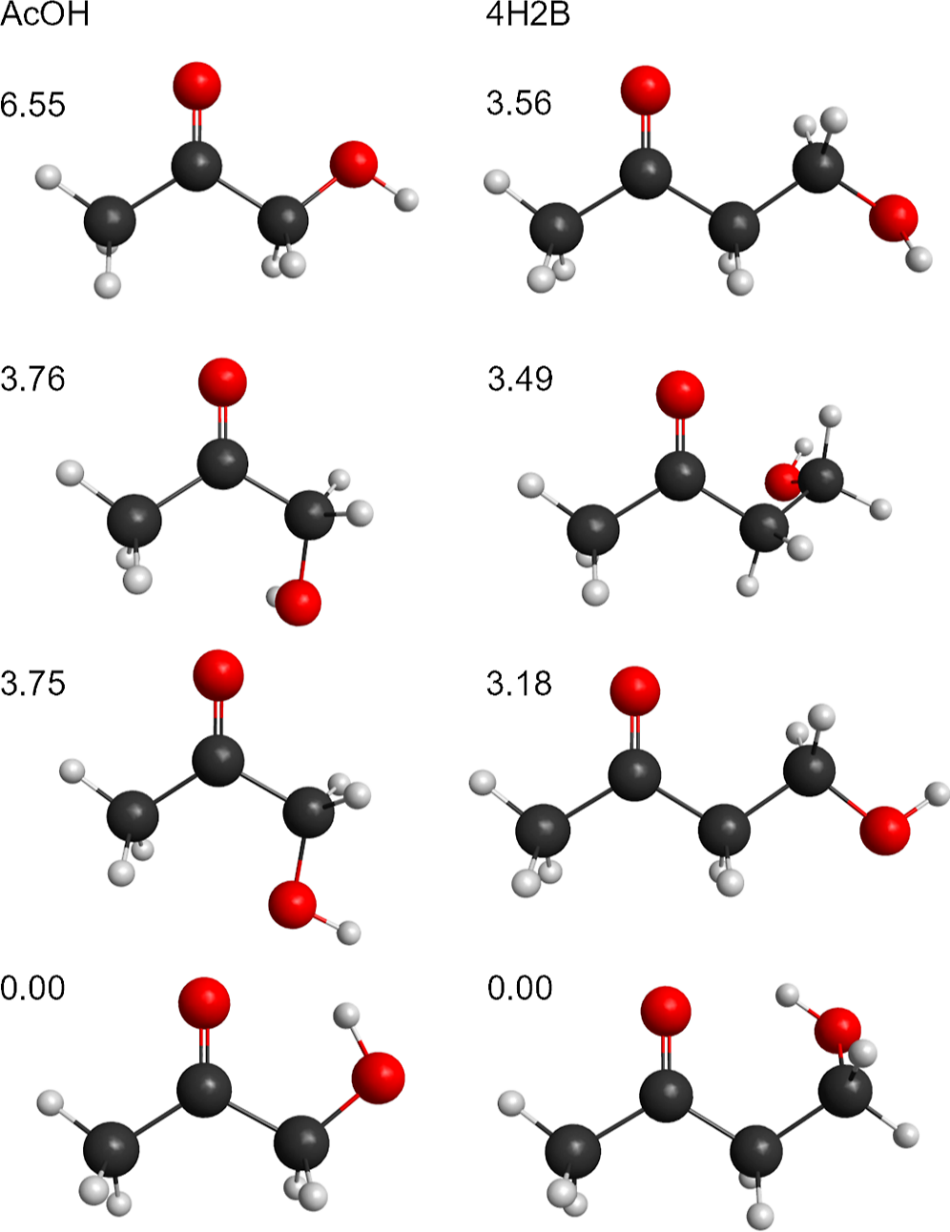
Low-energy conformers of AcOH (left) and 4H2B (right), calculated at the B3LYP/cc-pVDZ level. The zero-point corrected energy (kcal mol^–1^) of each conformer relative to the most stable cyclic H-bonded structures is indicated.

Preliminary calculations at the B3LYP/cc-pVDZ level of theory were performed to assess the possible relevance of different hydroxyketone conformers. Four low-energy conformers were identified for each molecule, distinguished primarily by rotations about the α C–C bond and the C–O bond. The optimized geometries are shown in Figure 4. The most stable hydroxyketone conformers are distinguished by the presence of an intramolecular hydrogen bond, resulting in cyclic five and six membered structures for AcOH and 4H2B, respectively. For both hydroxyketones, the Boltzmann population distribution is dominated at all temperatures (>98%) by the intramolecular H-bonded conformers, which are at least 3 kcal mol^–1^ lower in energy than the next lowest conformer. Higher energy conformers were assumed to play no significant role in the reactions with CH_2_OO and were neglected in subsequent calculations.

**Figure 5:**
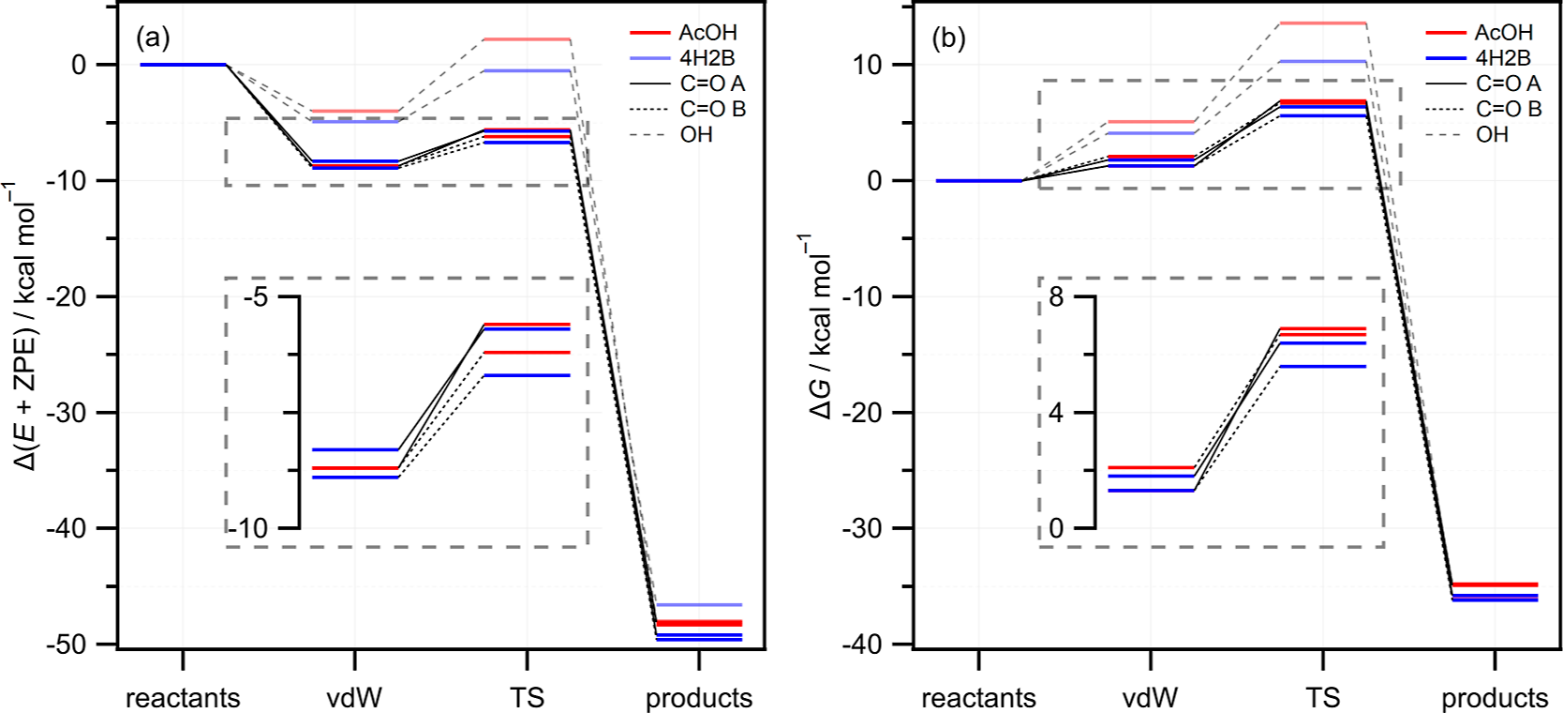
CBS-QB3 energy and free-energy profiles for the CH_2_OO + AcOH (red) and 4H2B (blue). (a) Δ(*E* + ZPE) at 0 K. (b) Δ*G* at 298 K for both reactions. Solid lines connect stationary points for cycloaddition A, dotted lines for cycloaddition B, and dashed lines for 1,2-addition at the OH group. The inset shows a magnified view of the entrance channel van der Waals complex (vdW) and the transition state (TS) for the cycloaddition pathways.

Hydroxyketones can react with CH_2_OO as either carbonyls or alcohols. Reaction at the carbonyl site occurs via a 1,3-dipolar cycloaddition, leading to the formation of a five membered cyclic trioxolane, or SOZ. Reaction at the hydroxyl group can occur via a 1,2-addition (or insertion) mechanism, leading to a substituted hydroperoxide. The overall zero-point corrected energy [Δ(*E* + ZPE) at 0 K] and Gibbs free energy (Δ*G*° at 298 K) profiles are shown in Figure 5. The reactions are similarly exoergic with products lying at values of Δ(*E* + ZPE) ≈ −50 kcal mol^–1^ (Δ*G*° ≈ −35 kcal mol^–1^). The entrance channels of both reactions support van der Waals complexes that are bound by 4–9 kcal mol^–1^, while the TSs have energies that are generally below the separated reactants. On the free energy surface, all entrance channel complexes and TSs are higher in energy than the reactants.

**Figure 6:**
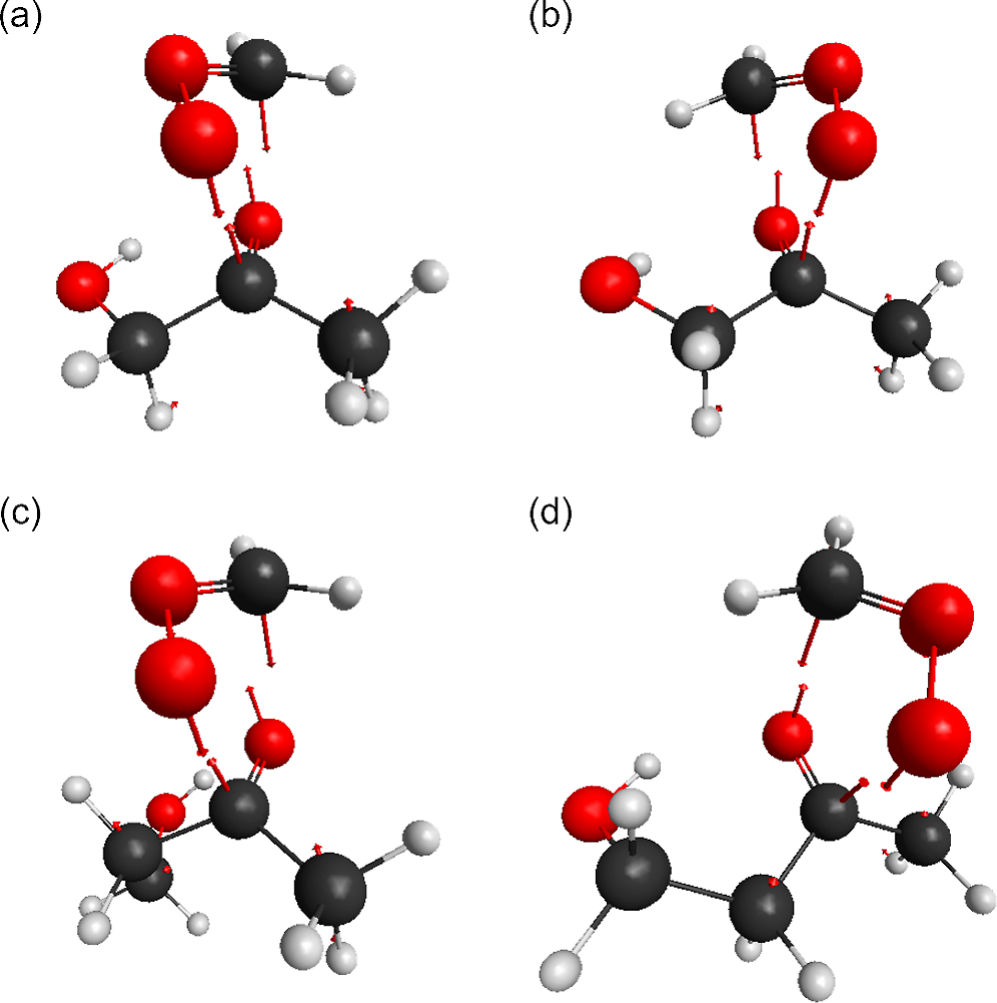
Optimized TS geometries for the 1,3-dipolar cycloaddition reactions of CH_2_OO with AcOH (a,b) and 4H2B (c,d). The orientation of the central O atom of CH_2_OO oriented toward (a,c) or away (b,d) from the hydroxyl group of the hydroxyketone identifies cycloaddition A or B, respectively. Red arrows indicate displacement vectors along the reaction coordinate.

Cycloaddition at the carbonyl site of each hydroxyketone can occur via two near-equivalent pathways that differ in the orientation of the CH_2_OO with respect to the OH group of the hydroxyketone. In pathway A, the central O atom of CH_2_OO is oriented toward the hydroxyalkyl side at TS_A_, while pathway B has the central O oriented toward the methyl side of the hydroxyketone at TS_B_. The optimized TS_A_ and TS_B_ geometries are shown in Figure 6. Unsurprisingly, the energies and free energies are similar. The free-energy barriers for the A and B pathways of the AcOH reaction differ by only 0.2 kcal mol^–1^, with a difference of 0.8 kcal is found for the 4H2B reaction, slightly favoring TS_B_. The larger energy difference for 4H2B arises because of a geometrical distortion of the hydroxyethyl group out of the plane, away from the attacking CH_2_OO, in TS_A_ while the carbon backbone maintains planarity in TS_B_. The TS free energies for reaction at the OH site of the hydroxyketones are significantly higher than those for the cycloaddition reactions (almost 7 kcal mol^–1^ for the AcOH reaction and ∼4 kcal mol^–1^ higher for the 4H2B reaction), as shown in Figure 5 and Table 2.

## Discussion

The CH_2_OO + AcOH and CH_2_OO + 4H2B reactions are found to have almost identical rate constants across the 275–335 K temperature range studied experimentally, as summarized in Table 1. At room temperature (∼295 K), the rate constants for both reactions are 1.1 × 10^–12^ cm^3^ s^–1^, which is greater than the reactions of simple ketones by around a factor of 2. The rate constant for the CH_2_OO + acetone (Ac) reaction has been measured by various groups, with values in the range *k*_Ac_ = (2.3–4.8) × 10^–13^ cm^3^ s^–1^ reported,^11,12,19−22^ where the range is likely a consequence of the reaction pressure dependence. Measurements at the high-*P* limit have a weighted average value *k*_Ac_ = 4.3 × 10^–13^ cm^3^ s^–1^.^11,12,21,22^ The rate constant for the CH_2_OO + methylethylketone (MEK) reaction is slightly larger than that of Ac with a value *k*_MEK_ = 6.4 × 10^–13^ cm^3^ s^–1^.^70^ The room temperature rate constants for the hydroxyketone reactions are much closer to that of acetaldehyde (MeCHO), for which rate constants in the range *k*_MeCHO_ = (1.0–1.7) × 10^–12^ cm^3^ s^–1^ have been reported.^19,20,23^ The CH_2_OO + R_1_R_2_CO reactions also all show similar negative temperature dependences.

The reactivity of the hydroxyketones toward CH_2_OO is increased relative to aliphatic ketones, although the effect is largely insensitive to whether the OH is at the α or β position. The OH group provides an additional site for reaction via the 1,2-addition mechanism that has been characterized for alcohols.^28,29,71,72^ In general, alcohols tend to react with CH_2_OO much more slowly than carbonyls. For example, the rate constant for the CH_2_OO + methanol (MeOH) reaction at room temperature is *k*_MeOH_ = 1.2 × 10^–13^ cm^3^ s^–1^.^28,29^ The increased rate constant for the hydroxyketones relative to acetone is likely due to enhancement of the 1,3-dipolar cycloaddition mechanism rather than being due to the presence of an additional reaction pathway. We note that synergistic rate constant increases arising from the presence of different functional groups have been demonstrated in other CI reactions. For example, 3-aminopropanol reacts with acetaldehyde oxide, CH_3_CHOO, in a concerted double hydrogen atom transfer step, where both the amine and hydroxyl functional groups interact with the CI simultaneously, significantly faster than simple amines or alcohols.^27,73^ Additionally, acetylacetone (AcAc), which exists predominantly as its enolone tautomer, has C=O, OH, and C=C sites for reaction with CH_2_OO. The CH_2_OO + AcAc reaction is twice as fast as CH_2_OO + Ac at room temperature (*k*_AcAc_ = 8.0 × 10^–13^ cm^3^ s^–1^) and shows a weak temperature dependence (−*E*_a_/*R* = 460 K) which can be explained in part by the existence of competitive pathways for reaction at both the C=O and C=C sites, where ab initio calculations find similar Δ^⧧^*G*° values at 298 K.^11,12^ Rate constants for CH_2_OO + alkene reactions are generally much smaller than that for carbonyls^74^ and show a positive temperature dependence. For the AcAc reaction, it appears that the adjacent carbonyl group may enhance the reactivity at the C=C site.

The experimental kinetics observations are supported by the ab initio calculations, which show the presence of relatively stable entrance channel complexes followed by TS barriers that are in most cases submerged relative to the reactants, consistent with the negative temperature dependences. Additionally, no dependence on total pressure was observed in the range 80–120 Torr of N_2_. A two-step mechanism for either the cycloaddition or 1,2-addition reactions can be written as
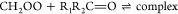
R1
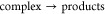
R2where the products are either SOZs or hydroperoxide species (see the Supporting Information). In the high-*P* limit, equilibrium is established for reaction R1, and the overall rate constant can be represented as *k* = *k*_2_*K*_1_, where *K*_1_ = *k*_1_/*k*_–1_. From the perspective of transition-state theory, the magnitude of the overall experimental rate constant is largely determined by the standard free energy of activation Δ^⧧^*G*° at the TS. The results of the CBS-QB3 ab initio calculations are summarized in Table 2 and Figure 5. Free-energy barriers for the cycloaddition reactions at the carbonyl are calculated to be broadly similar (∼+6 kcal mol^–1^), and analysis of the temperature dependence of the rate constants results in values that are in good agreement (see Table 1). In contrast, the calculated free-energy barriers for reaction at the hydroxyl group are significantly higher (>10 kcal mol^–1^). For comparison, the free energy of activation for CH_2_OO reacting with methanol is calculated to be Δ*G*° = +9.1 kcal mol^–1^ at the same CBS-QB3 level of theory (see Table 2). That is, the energy barrier for reaction at the OH position is even higher in the hydroxyketones than that in a simple alcohol like methanol, for which the observed rate constant is an order of magnitude lower. The higher energy barrier is consistent with the disruption of the intramolecular H-bond in the minimum energy structure of hydroxyketones required for reaction at the hydroxyl group. It is only the slightly higher-energy conformers, as shown in Figure 4, that can take part in the 1,2-addition with CH_2_OO. Consequently, we conclude that the dominant reaction between CH_2_OO and the hydroxyketones is the 1,3-dipolar cycloaddition at the C=O reaction site. The presence of the hydroxyl group on either the α or β carbon appears to have no significant effect on the observed rate constants, consistent with the similarity of the calculated free-energy barriers.

**Figure 7:**
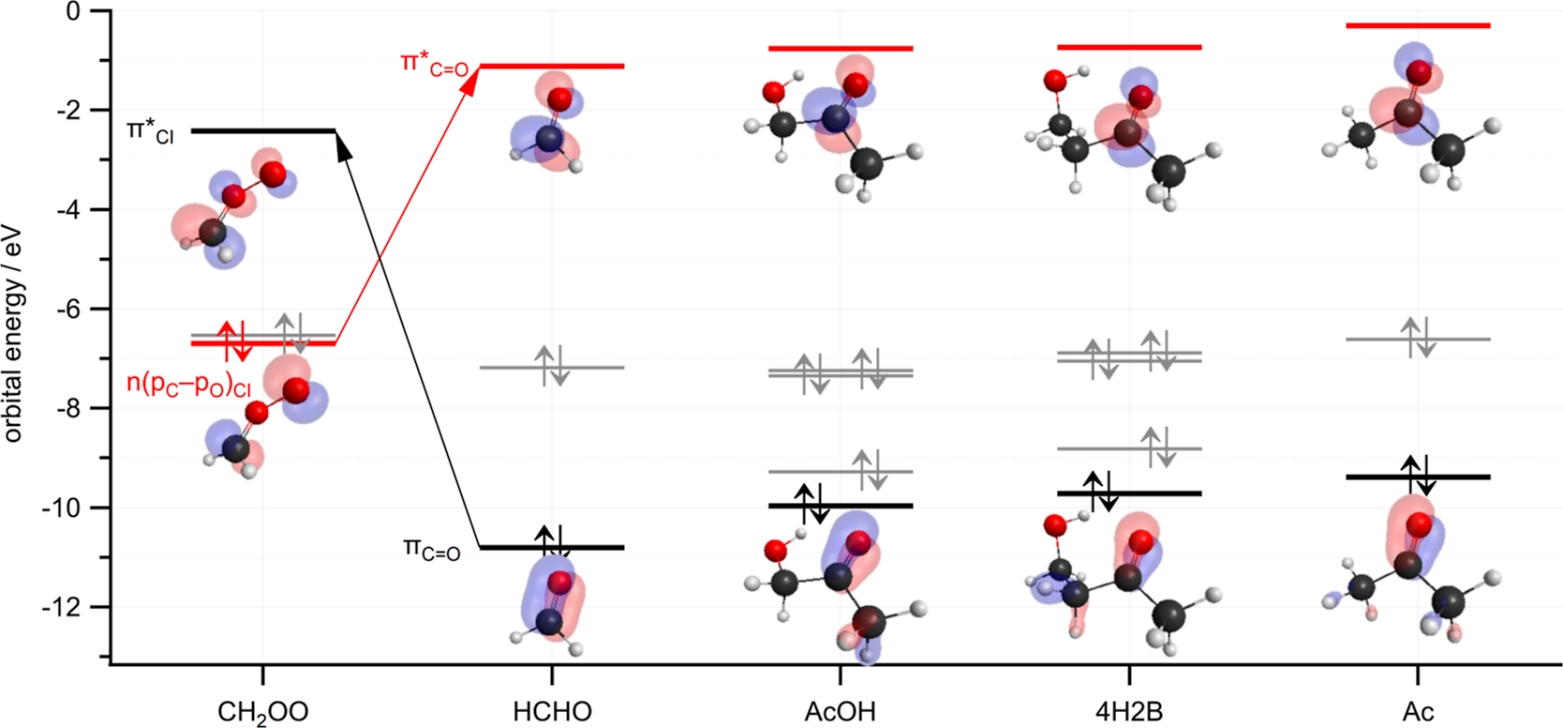
Frontier orbital energies calculated at the B3LYP/cc-pVDZ level for CH_2_OO and the carbonyls formaldehyde, acetone, and the hydroxyketones AcOH and 4H2B. Red: A orbitals (nonbonding *n*(*p*_C_–*p*_O_) for CH_2_OO, π* LUMO for carbonyls), black: S orbitals (π bonding orbitals of carbonyls, π* antibonding for CH_2_OO), and gray: nonbonding (*n*_O_ orbitals).

Previously,^11^ we have attempted to rationalize the reactivity trends in 1,3-dipolar cycloaddition reactions between CH_2_OO and carbonyl compounds (R_1_R_2_CO, where R1 and R2 are alkyl or acyl substituents) using a FMO theory approach.^13−16^ Starting with the model of symmetry-allowed orbital interactions developed by Sustmann,^13^ the cycloaddition between carbonyl compounds and CH_2_OO primarily involves interactions between the out-of-plane π and π* orbitals of CH_2_OO and R_1_R_2_CO. The dominant interaction is between the occupied nonbonding *n*(*p*_C_–*p*_O_) molecular orbital of the electron-rich species CH_2_OO (the 1,3-dipole) and the lowest unoccupied π* molecular orbital of the electron-deficient carbonyl, R_1_R_2_CO (the dipolarophile), with energy gap |Δ*E*_A_|. The energy gap |Δ*E*_S_| between the occupied carbonyl π orbital and the unoccupied π* orbital of CH_2_OO is, in general, larger and makes a smaller contribution to the reactivity. The energies of the carbonyl FMOs are affected by the electron-donating or electron-withdrawing nature of the substituents R_1_ and R_2_. EDGs on the carbonyl raise the energy of the orbitals, increasing the magnitude of |Δ*E*_A_|, leading to a decreased reactivity. In contrast, EWGs lower the orbital energies and have the opposite effect on reactivity. The orbital interactions are illustrated in Figure 7, which shows the FMOs of CH_2_OO, and the carbonyls formaldehyde (HCHO), AcOH, 4H2B, and acetone (Ac), calculated at the B3LYP/cc-pVDZ level of theory.

The EWG or EDG character of the R_1_ and R_2_ substituents can be represented by Hammett substituent constants, σ_m_ and σ_p_, where the subscripts refer to substitution at the meta/para position of benzoic acid.^18^ Positive values indicate EWG character and increased reactivity, while negative values indicate EDG character and reduced reactivity. Among the carbonyls HCHO, AcOH, 4H2B, and Ac, the substituents and their Hammett constants are H (σ_m_ = σ_p_ = 0), CH_3_ (σ_m_ = −0.07, σ_p_ = −0.17), and CH_2_OH (σ_m_ = σ_p_ = 0), while values for CH_2_CH_2_OH are unknown. Methyl is electron-donating, raising the energies of the frontier orbitals while the others have no significant effect. The effect of the electron-donating methyl groups on the calculated FMO energies is evident in Figure 7 and Table 3. Relative to H, each methyl group substituent increases the carbonyl FMO energies by ∼0.4 eV. Based on the calculated orbital energies in Table 3, it is likely that CH_2_CH_2_OH is marginally more electron-donating than CH_2_OH due to the presence of the additional methylene group between the hydroxyl and the carbonyl. Although the experimental rate constants were found to be indistinguishable, accounting for the possible systematic error in the UV absorption cross section of AcOH described above would lead to a 10% increase in *k*_AcOH_, in line with the expectations of the orbital analysis.

**Table 3:** B3LYP/cc-pVDZ Calculated Orbital Energies (eV) of HCHO, AcOH, 4H2B, and Ac^a^

	HCHO	AcOH	4H2B	Ac
π*	–1.116	–0.762	–0.735	–0.299
|Δ*E*_A_|	5.578	5.932	5.959	6.395
π	–10.803	–9.959	–9.714	–9.388
|Δ*E*_S_|	6.980	7.538	7.293	6.966

a CH_2_OO π* *E* = −2.422 eV, *n*(*p*_C_–*p*_O_) *E* = −6.694 eV. |Δ*E*_A_| is the magnitude of the energy difference between the occupied *n*(*p*_C_–*p*_O_) orbital of CH_2_OO and unoccupied π* of the carbonyl (the dominant interaction). |Δ*E*_S_| is the magnitude of the difference between the unoccupied π* orbital of CH_2_OO and the occupied π orbital of the carbonyl. The |Δ*E*_A_| interaction is always smaller.

**Figure 8:**
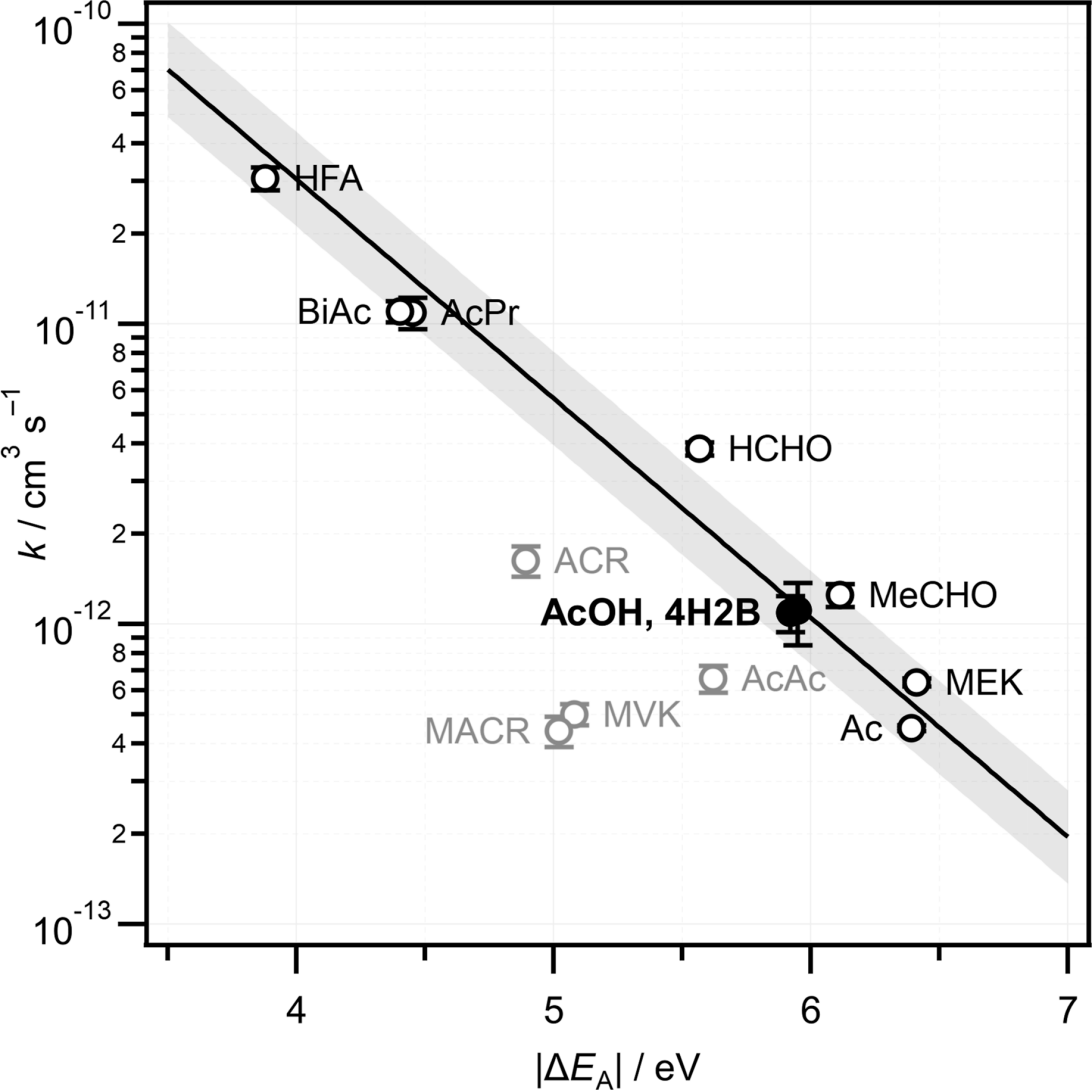
Inverse correlation between reported experimental rate constants at room temperature for the reactions of CH_2_OO with a series of carbonyl compounds and the energy gap |Δ*E*_A_| between the π* orbital of the carbonyl and the *n*(*p*_C_–*p*_O_) orbital of CH_2_OO. Orbital energies were calculated at the B3LYP/cc-pVDZ level. A linear fit is shown, where the shaded area represents 1σ prediction bands. Experimental rate constants are drawn from various sources, see the text for details. Reactions involving α,β-unsaturated carbonyls (gray) were excluded from the fit.

Previously, we showed that the relationship between observed rate constants and calculated orbital energy gaps could be used quantitatively.^11^Figure 8 shows an updated plot of ln *k* against the magnitude of the orbital energy gap |Δ*E*_A_|, for a range of CH_2_OO + R_1_R_2_CO reactions. Experimental rate constants at room temperature have been obtained from various sources^11,12,19,22,25,26,70,75,76^ and are compiled in Table S2 of Supporting Information. The data set includes reactions of CH_2_OO with ketones, α-diketones, aldehydes, and α,β-unsaturated enones and enals. Where more than one experimental value is available, the weighted average rate constant is used. Orbital energies have been calculated at the B3LYP/cc-pVDZ level, rather than M06-2X/aug-cc-pVTZ as used previously.^11^ The effect of the electron-donating or electron-withdrawing character of the R_1_ and R_2_ substituents on the orbital energy gaps and rate constants leads to a strong negative linear correlation. The fastest reaction is with HFA, which has strongly electron-withdrawing substituents (R_1_ = R_2_ = CF_3_, σ_m_ = 0.43, σ_p_ = 0.54), while the slowest reaction is with acetone, which has electron-donating substituents (R_1_ = R_2_ = CH_3_). For species with R_1_ ≠ R_2_, such as acetaldehyde (R_1_ = H, R_2_ = CH_3_) or the hydroxyketones, the effect is additive. The α,β-unsaturated carbonyls such as MVK, MACR, ACR, and the enolone form of AcAc are shown in Figure 8 but clearly deviate from the trend and are not included in the fit. The |Δ*E*_A_| values for these species imply that the reactions with CH_2_OO should be much faster than observed experimentally,^11,12,75,76^ suggesting that delocalization of the π system reduces the reactivity of the carbonyl. Further work is required to explain the reactivity of α,β-unsaturated carbonyls toward CH_2_OO, which deviate from the trend.

### Atmospheric Implications

The major reactive sink for hydroxyketones in the atmosphere is reaction with OH radicals. Rate constants for reaction with OH are 2.0 × 10^–12^ exp(−320/*T*) cm^3^ s^–1^ and 1.3 × 10^–12^ exp(−400/*T*) cm^3^ s^–1^ for AcOH and 4H2B, respectively.^47,51^ Typical lifetimes for both hydroxyketones reacting with OH radical are ∼3 days in the troposphere.^38,39,43,44,49,51^ Comparatively, photolysis has a minor contribution with lifetimes of ∼12–14 days for AcOH and 26 days for 4H2B.^43,51^ Temperature-dependent lifetimes for AcOH and 4H2B were estimated using typical average tropospheric concentrations for CH_2_OO and OH. The average CH_2_OO concentration was assumed to be 2 × 10^4^ cm^–3^, although concentrations as high as 1 × 10^5^ cm^–3^ have been reported.^3,77^ The average concentration of OH was estimated to be 5 × 10^6^ cm^–3^ during the day and 2 × 10^5^ cm^–3^ at night.^78,79^ At 295 K, the hydroxyketone reactions with CH_2_OO are insignificant, with estimated lifetimes >500 days. However, the CH_2_OO reactions show a strong negative *T* dependence and may become relatively more important at lower temperatures. While the hydroxyketone loss rates due to CH_2_OO increase at lower temperature, with lifetimes of ∼80 days at 220 K, OH remains the most important reactive sink for both hydroxyketones across the temperature range, even at night when OH concentrations are markedly lower.

## Conclusions

The kinetics of the CH_2_OO + AcOH and CH_2_OO + 4H2B reactions were measured across the temperature range 275–335 K using a flash photolysis, transient absorption spectroscopy technique. The temperature-dependent bimolecular rate constants are *k*_AcOH_ = (4.3 ± 1.7) × 10^–15^ exp[(1630 ± 120)/*T*] and *k*_4H2B_ = (3.5 ± 2.6) × 10^–15^ exp[(1700 ± 200)/*T*]. Complementary ab initio calculations confirm that both reactions proceed via 1,3-dipolar cycloaddition at the carbonyl to form cyclic SOZs, while reaction at the hydroxyl group via 1,2-addition is insignificant. The increased reactivity of hydroxyketones relative to acetone can be understood from a FMO theory approach, wherein the cycloaddition involves an interaction between the occupied *n*(*p*_C_–*p*_O_) orbital of the CH_2_OO and the unoccupied π* orbital of the carbonyl. Electron-donating or electron-withdrawing substituents increase or decrease the π* orbital energy. Alkyl substituents are electron-donating, which decreases reactivity, while hydroxyalkyl substituents are, like H, neither electron-donating nor electron-withdrawing. A strong inverse correlation is found between the logarithm of the rate constants and the orbital energy gap for a range of R_1_R_2_CO species. The reactions of CH_2_OO with AcOH and 4H2B are unlikely to be significant in the troposphere, where reaction with hydroxyl radicals and photolysis control the hydroxyketone lifetimes.

## Supporting Information Available

Description of the calibration procedure; FT-IR spectra of AcOH and 4H2B; UV absorption spectra of AcOH and 4H2B; concentration calibration plots; description of the kinetic model; example transient absorption spectra and [CH_2_OO] time profile; global pseudo-first-order plots; plot of ln(*k*/*T*^2^) against 1/*T*; experimental values of enthalpy, entropy, and Gibbs free energy of activation; pressure dependence of CH_2_OO loss rates; compilation of room temperature rate constants for CH_2_OO + R_1_R_2_CO reactions; and CBS-QB3 Cartesian coordinates and energies of the reactants, complexes, TSs, and products (PDF)
