# Supplementary material for: Temperature-Dependent Kinetics of the Reactions of the Criegee Intermediate CH2OO with Hydroxyketones
Source: J Phys Chem A. 2024 Mar 1;128(10):1880–91. doi: 10.1021/acs.jpca.4c00156 (PMC10945482; doi:10.1021/acs.jpca.4c00156)
Supplement: Supplementary file 1 — jp4c00156_si_001.pdf [file jp4c00156_si_001.pdf]

Supporting Information: Temperature-Dependent Kinetics of the Reactions of the Criegee Intermediate CH_2OO with Hydroxyketones

Zachary A. Cornwell,[1] Jonas J. Enders,[1] Aaron W. Harrison,[2] and Craig Murray[*,1]

1. *Department of Chemistry, University of California, Irvine, Irvine CA 92697, USA*

2. *Department of Chemistry, Austin College, Sherman, TX 75090, USA*

Reactant Concentration Measurements

The AcOH and 4H2B samples were used as provided by the manufacturers, with reported purities of 90% and 95%, respectively. IR spectra of the headspace above liquid samples of both hydroxyketones were recorded in a 10 cm cell with ZnSe windows in a JASCO 4700 FT-IR spectrometer to identify any impurities present. The spectra are shown in Figure S1. The AcOH spectrum is the same as the that obtained from the PNNL database,[1] with no obvious features present that could be attributed to impurities. To the best of our knowledge, the gas-phase spectrum of 4H2B has not been reported in the literature, but it appears very similar to that of AcOH, although the absorbance is weaker as a result of its lower vapor pressure.

The concentrations (cm^{-3}) of the hydroxyketone reactants present in the flow reactor during measurements can be estimated based on reported vapor pressures[2,3] and the gas flow rates using the equation

$$[\mathrm{X}]_{\mathrm{est}} = \chi F P_{\mathrm{tot}} \frac{N_{\mathrm{A}}}{RT}$$

where χ is the mole fraction of X in the X/N_2 flow, F is its fractional contribution to the total gas flow, P_{tot} is the total pressure (Torr), N_{A} is the Avogadro constant (6.022×10^{23} mol^{-1}), T is the reactor temperature (K), and R is the gas constant (62.364×10^{3} cm^{3} $Torr^{-1}$ mol^{-1} K^{-1}). The actual concentrations $[\mathrm{X}]_{\mathrm{exp}}$ are determined from absorption spectra recorded in the 275–298 nm range using a pulsed LED nominally centered at 280 nm under identical conditions to those used in the kinetics measurements. Figure S2 shows reported absorption spectra for AcOH and 4H2B.[4–7] The JPL recommendation was used for AcOH. The calibration plots of $[\mathrm{X}]_{\mathrm{exp}}$ versus $[\mathrm{X}]_{\mathrm{est}}$ shown in Figure S3 and Figure S4 were used to determine temperature-independent scaling factors that allow the concentration estimates to be corrected.

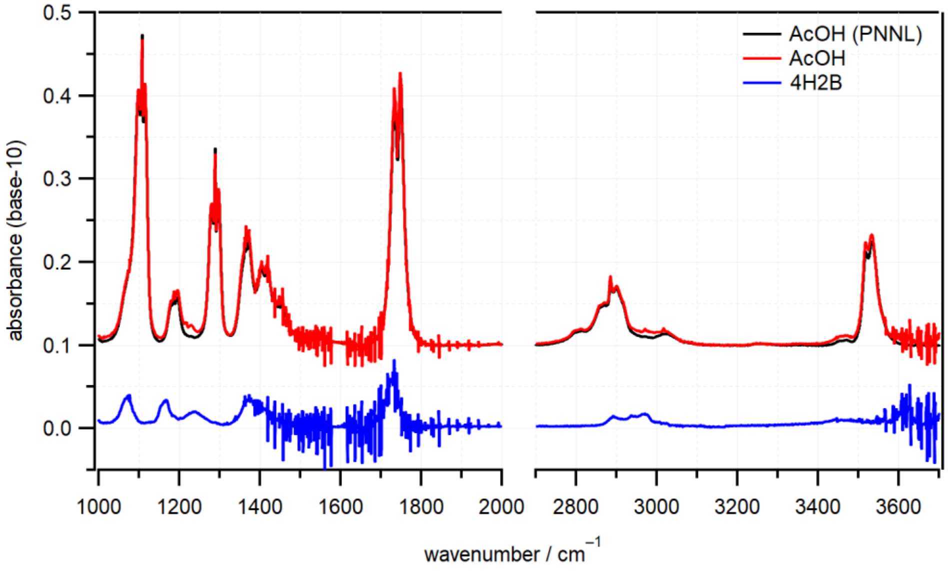

Figure S1 Headspace FT-IR spectra of AcOH (red) and 4H2B (blue). Also shown is the AcOH spectrum (black) obtained from the PNNL IR spectral database.[1]

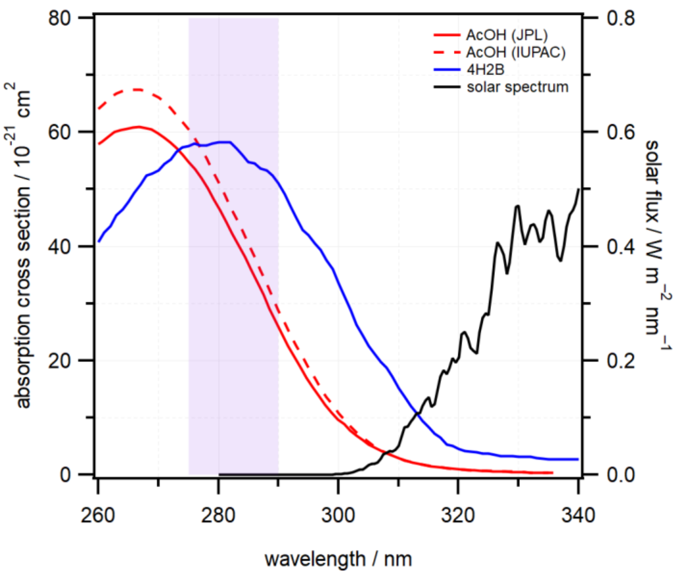

Figure S2 UV absorption spectra of AcOH (red) and 4H2B (blue). The shaded area indicates the wavelength range covered by the 280 nm LED used for concentration measurements. Solar flux (black) is also shown.

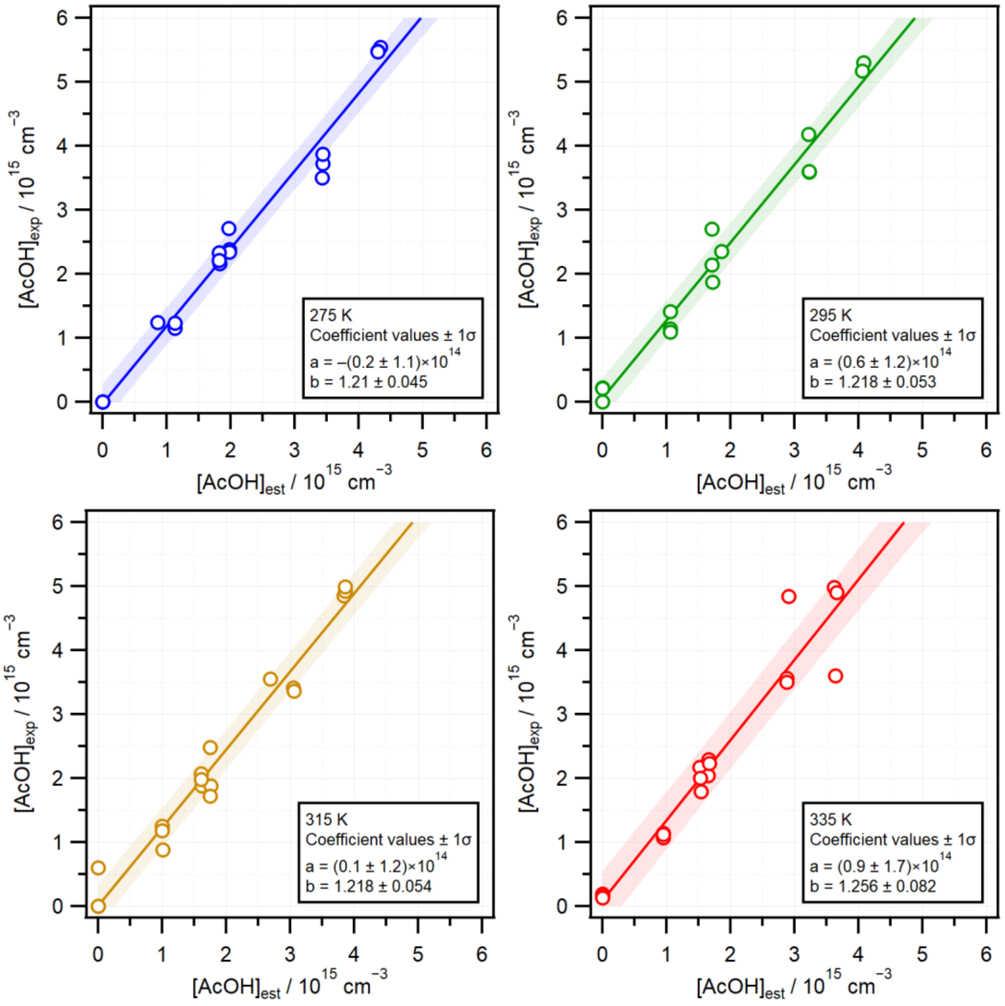

Figure S3 Calibration plots of experimental versus estimated AcOH concentrations in the range 275–335 K.

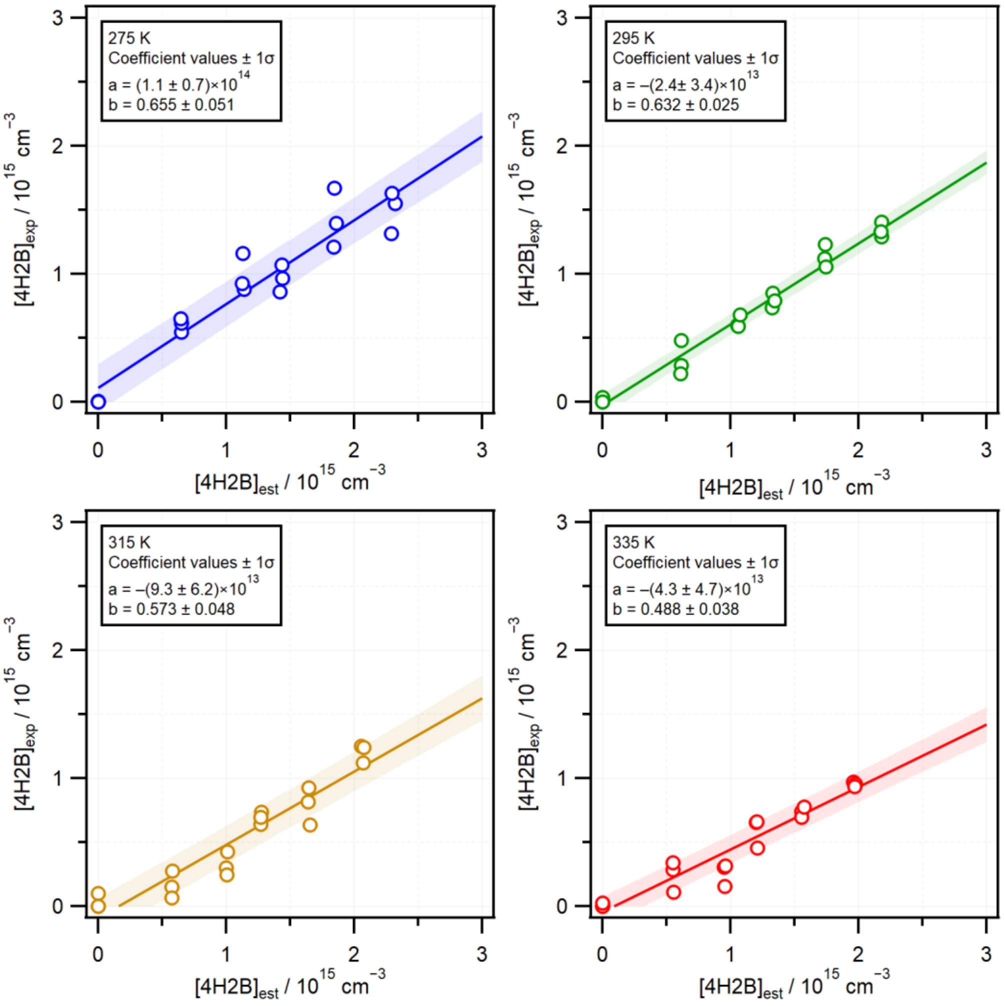

Figure S4 Calibration plots of experimental versus estimated 4H2B concentrations in the range 275–335 K.

Kinetics Measurements

Examples of typical transient absorption spectra, with and without AcOH present, are shown in Figure S5(a) and (b). The spectral fits used to determine [CH_2OO] at each time delay are superimposed. The resulting time-dependent CH_2OO concentrations are shown in Figure S5(c). The time-dependent concentrations are fit to the integrated rate equation

$$[\mathrm{CH_2OO}]_t = \frac{k_{\mathrm{loss}}[\mathrm{CH_2OO}]_0}{k_{\mathrm{loss}}\exp(k_{\mathrm{loss}}t) - 2k_{\mathrm{self}}[\mathrm{CH_2OO}]_0[1-\exp(k_{\mathrm{loss}}t)]}$$

To extract k_{loss}, a pseudo-1st order rate constant that accounts for background losses and reaction with the hydroxyketone X

$$k_{\mathrm{loss}} = k_{\mathrm{bgd}} + k_{\mathrm{X}}[\mathrm{X}]$$

k_{self} is the self-reaction rate constant, which is held fixed at 7.8×10^{-11} cm^3 s^{-1}.[8]

Figure S6 and Figure S7 show the pseudo-1st order plots of CH_2OO loss rates as a function of the AcOH and 4H2B concentrations, respectively, at temperatures in the range 275–335 K.

Figure S8 shows plots of ln (k/T_2) against $1/T$ for the CH_2OO + AcOH and CH_2OO + 4H2B reactions that are used to obtain the standard entropy, enthalpy, and Gibbs energy of activation, summarized in Table S1, according to the equation

$$k = \frac{k_B^2}{hp^\circ}\exp\left(\frac{\Delta^\ddagger S^\circ}{R}\right)T^2\exp\left(-\frac{\Delta^\ddagger H^\circ}{RT}\right)$$

where p° is standard pressure of 10^5 Pa.

Figure S9 shows CH_2OO loss rates in the presence of AcOH and 4H2B as a function of total pressure.

Experimental rate constants for a range of CH_2OO + R_1R_2CO reactions at room temperature are compiled in Table S2.

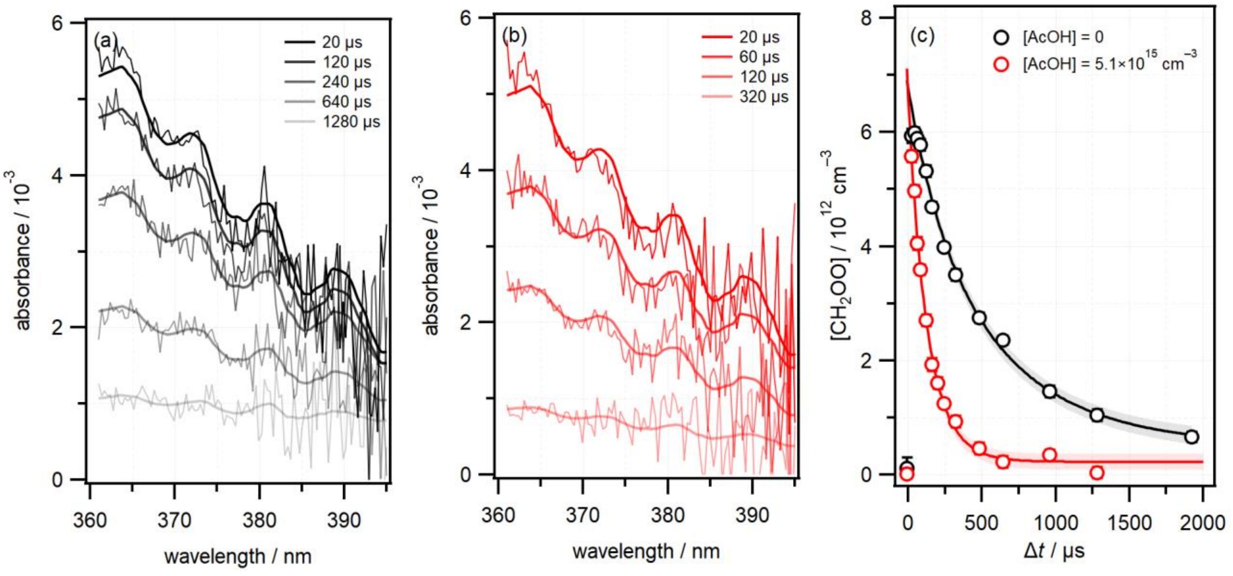

Figure S5 Broadband transient absorption spectra recorded at various time delays after photolysis at 295 K. (a) Spectra recorded in the absence of any additional reactive species, (b) spectra recorded with [AcOH = 5.1×10^{15} cm^{-3}. (c) [CH_2OO] time profiles resulting from fitting the experimental transient spectra.

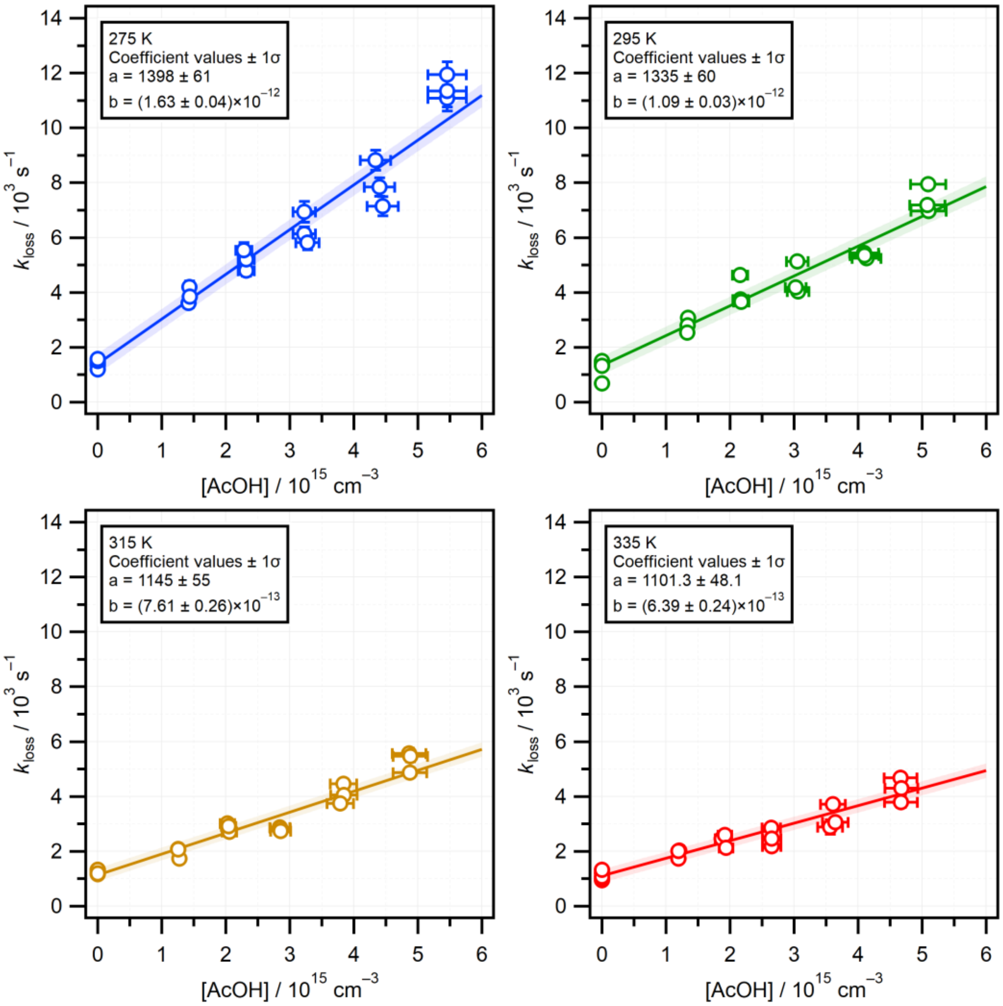

Figure S6 Global pseudo-1st order plots for the reaction of CH_2OO with AcOH in the temperature range 275–335 K. Three independent kinetic runs are compiled at each temperature.

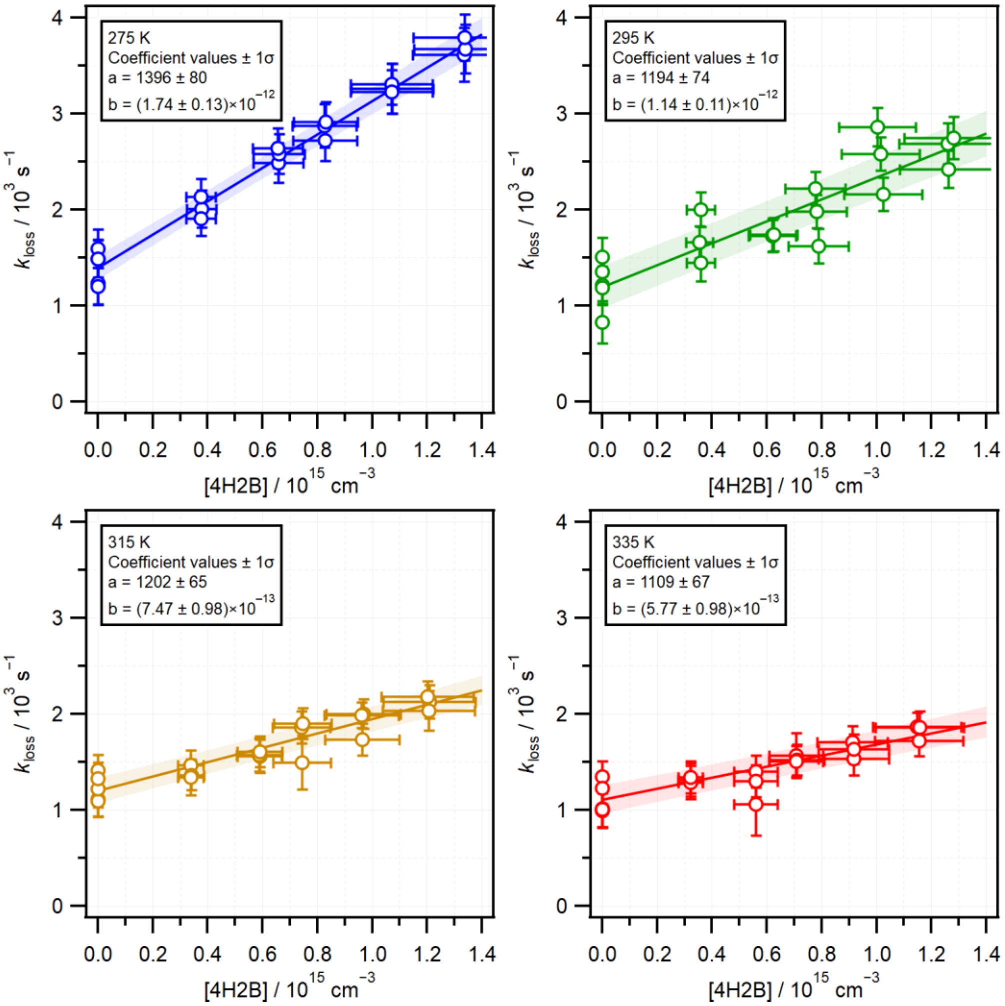

Figure S7 Global pseudo-1st order plots for the reaction of CH_2OO with 4H2B in the temperature range 275–335 K. Three independent kinetic runs are compiled at each temperature.

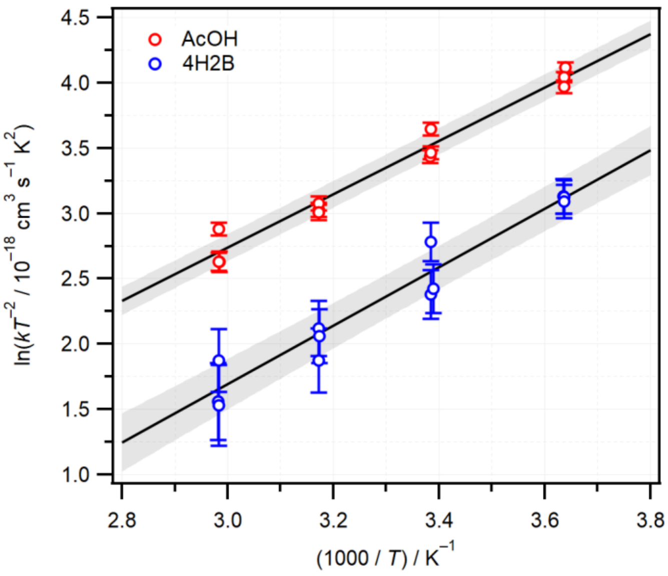

Figure S8 Plots of $\ln(k/T^2)$ against $1/T$ for the reaction of CH_2OO with AcOH (red) and 4H2B (blue), with 1σ statistical uncertainties. The AcOH data has been offset vertically for clarity. Solid black lines are linear fits with shaded areas representing 1σ prediction bands.

Table S1 Standard enthalpy, entropy, and Gibbs free energy changes (at 298 K) for the rate-determining TS derived from fits of the temperature-dependent rate constants shown in Figure S8. Uncertainties are 1σ statistical uncertainties from the fits.

	AcOH	4H2B
$\Delta^{\ddagger}H^{\circ}$ / kcal mol^{-1}	−4.06±0.06	−4.44±0.20
$\Delta^{\ddagger}S^{\circ}$ / cal K^{-1} mol^{-1}	−38.2±0.2	−39.5±0.8
$\Delta^{\ddagger}G^{\circ}$ / kcal mol^{-1}	+7.35±0.09	+7.33±0.31

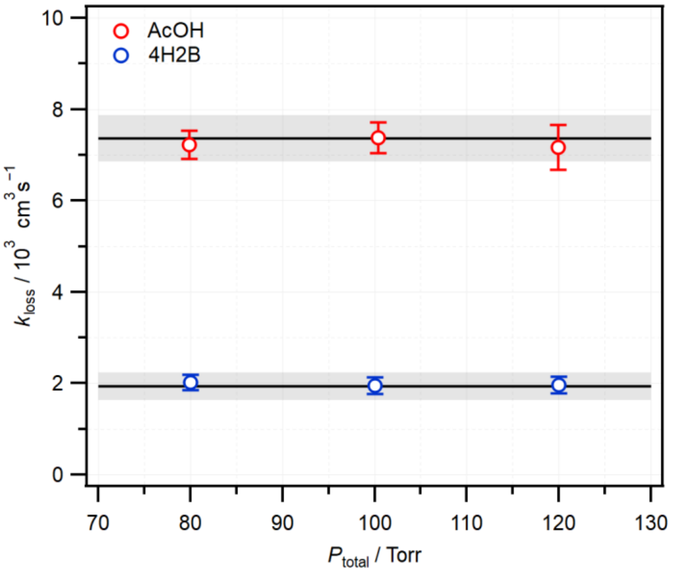

Figure S9 Pressure dependence of CH_2OO loss rates in the presence of AcOH (red, [AcOH] = $(5.1\pm0.3)\times10^{15}$ cm^{-3}) and 4H2B (blue, [4H2B] = $(8.0\pm1.1)\times10^{14}$ cm^{-3}) at 295 K. Total pressure was varied by changing only the flow rate of the N_2 buffer, with all other gas flows held constant. The solid lines indicate the average loss rates determined from the kinetics measurements. Shaded areas represent 1σ uncertainties.

Table S2 Experimental rate constants for CH_2OO + R_1R_2CO reactions at room temperature.

R_1R_2CO	R_1, R_2	$k_{R_1R_2CO}$ / 10^{-13} cm^3 s^{-1}	Reference
formaldehyde (HCHO)	H, H	41.1±2.5	Luo et al.[9]
		35.0±2.9	unpublished results
		**38.5±1.9**	**weighted mean**
acetaldehyde (MeCHO)	H, CH_3	17	Stone et al.[10]
		9.5±0.7*	Taatjes et al.[11]
		13±2	Elsamra et al.[12]
		16.1±1.4	unpublished results
		**15.1±1.1**	**weighted mean**
acetone (Ac)	CH_3, CH_3	2.3±0.3	Taatjes et al.[11]
		3.5±0.8	Elsamra et al.[12]
		3.4±0.9	Berndt et al.[13]
		4.7±0.1	Chhantyal-Pun et al.[14]
		4.8±0.4	Cornwell et al.[8]
		**4.5±0.1**	**weighted mean**
methyl ethyl ketone (MEK)	CH_3, C_2H_5	6.4±0.2	Debnath & Rajakumar[15]
hexafluoroacetone (HFA)	CF_3, CF_3	306±27	Taatjes et al.[11]
		333±63	Liu et al.[16]
		**306±27**	**weighted mean**
biacetyl (BiAc)	CH_3, CH_3CO	129±18	Cornwell et al.[8]
acetylpropionyl (AcPr)	CH_3, C_2H_5CO<br>C_2H_5, CH_3CO	109±13	Cornwell et al.[17]
acrolein (ACR)	H, $CH{=}CH_2$	16.3±1.9	Eskola et al.[18]
methacrolein (MACR)	H, $C(CH_3){=}CH_2$	4.4±0.1	Eskola et al.[18]
methyl vinyl ketone (MVK)	CH_3, $CH{=}CH_2$	5.0±0.4	Zhou et al.[19]
acetylacetone (AcAc)	CH_3, $CH{=}C(OH)CH_3$	8.0±0.7	Cornwell et al.[8]
hydroxyacetone (AcOH)	CH_3, CH_2OH	10.9±1.5	this work
4-hydroxy-2-butanone (4H2B)	CH_3, CH_2CH_2OH	11.1±2.6	this work

CBS-QB3 Cartesian Coordinates and Energies

CH_2OO

C	1.071833	-0.206302	0.000000
H	1.979866	0.382170	0.000000
H	1.020472	-1.289967	0.000000
O	0.000000	0.456082	0.000000
O	-1.178917	-0.187881	0.000000

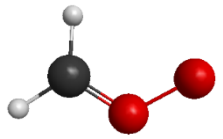

Temperature=	298.150000	Pressure=	1.000000
E(ZPE)=	0.030707	E(Thermal)=	0.034000
E(SCF)=	-188.623974	DE(MP2)=	-0.633244
DE(CBS)=	-0.062931	DE(MP34)=	-0.020139
DE(CCSD)=	-0.023792	DE(Int)=	0.019465
DE(Empirical)=	-0.029217		
CBS-QB3 (0 K)=	-189.343125	CBS-QB3 Energy=	-189.339832
CBS-QB3 Enthalpy=	-189.338887	CBS-QB3 Free Energy=	-189.367203

AcOH

C	-0.114880	-0.013932	0.190254
C	-0.205651	1.305489	-0.562794
O	-0.114420	-0.003127	1.403011
H	0.717710	1.411909	-1.155088
H	-1.034138	1.242669	-1.283459
C	-0.046257	-1.287604	-0.617032
H	-1.064419	-1.589529	-0.885326
H	0.511441	-1.149890	-1.546472
H	0.401741	-2.081240	-0.019873
O	-0.380460	2.388724	0.306833
H	-0.319888	2.010782	1.198679

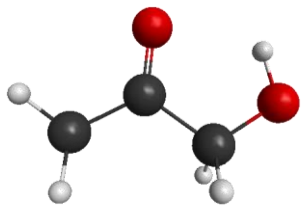

Temperature=	298.150000	Pressure=	1.000000
E(ZPE)=	0.087866	E(Thermal)=	0.093966
E(SCF)=	-266.911615	DE(MP2)=	-0.962079
DE(CBS)=	-0.095954	DE(MP34)=	-0.039062
DE(CCSD)=	-0.023692	DE(Int)=	0.030933
DE(Empirical)=	-0.047697		
CBS-QB3 (0 K)=	-267.961299	CBS-QB3 Energy=	-267.955199
CBS-QB3 Enthalpy=	-267.954255	CBS-QB3 Free Energy=	-267.990750

4H2B

C	-0.887083	0.146718	0.025771
O	-0.555288	1.297958	0.230064
C	0.141465	-0.966617	-0.106896
H	-0.216262	-1.856607	0.425238
H	0.185349	-1.241051	-1.169175
C	1.537497	-0.559019	0.371283
H	2.218274	-1.407860	0.275286
H	1.493119	-0.288957	1.436112
C	-2.343890	-0.241337	-0.110016
H	-2.497061	-0.889792	-0.977310
H	-2.651372	-0.811067	0.773586
H	-2.961147	0.652244	-0.192226
O	2.086048	0.492061	-0.402953
H	1.495079	1.244471	-0.269251

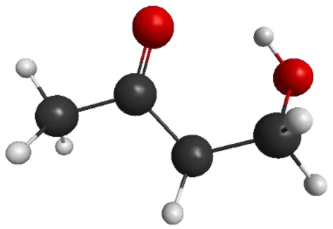

Temperature=	298.150000	Pressure=	1.000000
E(ZPE)=	0.116819	E(Thermal)=	0.123949
E(SCF)=	-305.959997	DE(MP2)=	-1.133090
DE(CBS)=	-0.111883	DE(MP34)=	-0.051410
DE(CCSD)=	-0.028729	DE(Int)=	0.036693
DE(Empirical)=	-0.056533		
CBS-QB3 (0 K)=	-307.188131	CBS-QB3 Energy=	-307.181001
CBS-QB3 Enthalpy=	-307.180056	CBS-QB3 Free Energy=	-307.219328

CH_2OO + hydroxyacetone (cycloaddition A)

vdW complex

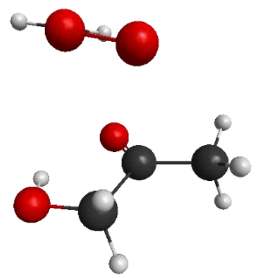

O	-1.943846	0.161700	-0.509288
O	-1.258839	-0.910367	-0.998113
O	0.475023	-0.045874	1.366835
O	0.946975	2.009624	-0.208290
C	1.026932	-0.349800	0.320258
C	1.477706	-1.752493	0.011971
C	-2.217044	0.186910	0.714043
C	1.382270	0.752725	-0.665233
H	1.385528	-1.972736	-1.052291
H	2.532753	-1.851374	0.292376
H	0.893172	-2.465058	0.591152
H	-1.924985	-0.641307	1.347746
H	-2.737123	1.076390	1.048089
H	2.474554	0.732502	-0.814030
H	0.911023	0.514483	-1.623651
H	0.691387	1.862385	0.715218

Temperature=	298.150000	Pressure=	1.000000
E(ZPE)=	0.120420	E(Thermal)=	0.131267
E(SCF)=	-455.548242	DE(MP2)=	-1.598011
DE(CBS)=	-0.158615	DE(MP34)=	-0.059126
DE(CCSD)=	-0.048125	DE(Int)=	0.050354
DE(Empirical)=	-0.076895		
CBS-QB3 (0 K)=	-457.318239	CBS-QB3 Energy=	-457.307392
CBS-QB3 Enthalpy=	-457.306447	CBS-QB3 Free Energy=	-457.355918

TS

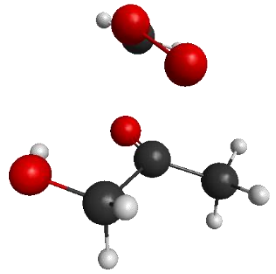

O	-1.648509	-0.626655	0.543211
O	-0.889455	0.302289	1.233512
O	-0.011844	0.103430	-1.258178
O	1.381586	-1.777733	-0.020030
C	0.625551	0.514102	-0.256998
C	0.814050	1.987252	0.004505
C	-2.040729	-0.189769	-0.575776
C	1.634883	-0.449427	0.361983
H	1.026300	2.181525	1.056949
H	1.661912	2.342496	-0.592091
H	-0.072270	2.544229	-0.296414
H	-2.110903	0.873243	-0.752979
H	-2.503318	-0.929489	-1.219607
H	2.630947	-0.113090	0.020508
H	1.611588	-0.375592	1.449813
H	0.899000	-1.706912	-0.856585

Temperature=	298.150000	Pressure=	1.000000
E(ZPE)=	0.121949	E(Thermal)=	0.130910
E(SCF)=	-455.533288	DE(MP2)=	-1.610819
DE(CBS)=	-0.159343	DE(MP34)=	-0.056205
DE(CCSD)=	-0.049326	DE(Int)=	0.050409
DE(Empirical)=	-0.076730		
CBS-QB3 (0 K)=	-457.313354	CBS-QB3 Energy=	-457.304393
CBS-QB3 Enthalpy=	-457.303448	CBS-QB3 Free Energy=	-457.347031

SOZ: (3-methyl-1,2,4-trioxolan-3-yl)methanol

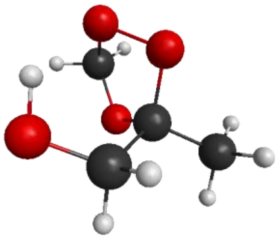

O	1.513675	0.080953	0.723160
O	0.246415	-0.499643	1.160680
O	0.329768	-0.533594	-1.099978
O	-0.218300	2.113834	-0.170074
C	-0.575511	-0.332562	-0.013955
C	-1.627872	-1.425556	0.025582
C	1.632385	-0.510474	-0.560741
C	-1.173516	1.080219	-0.072106
H	-2.292229	-1.294337	0.881716
H	-2.221020	-1.391045	-0.889624
H	-1.140344	-2.397617	0.095868
H	2.029846	-1.528859	-0.477711
H	2.284700	0.140401	-1.146505
H	-1.800836	1.149913	-0.964030
H	-1.816785	1.201658	0.811735
H	0.451287	1.957724	0.505573

Temperature=	298.150000	Pressure=	1.000000
E(ZPE)=	0.126594	E(Thermal)=	0.134726
E(SCF)=	-455.615126	DE(MP2)=	-1.608645
DE(CBS)=	-0.159451	DE(MP34)=	-0.053944
DE(CCSD)=	-0.043034	DE(Int)=	0.049693
DE(Empirical)=	-0.077241		
CBS-QB3 (0 K)=	-457.381153	CBS-QB3 Energy=	-457.373021
CBS-QB3 Enthalpy=	-457.372077	CBS-QB3 Free Energy=	-457.413554

CH_2OO + hydroxyacetone (cycloaddition B)

vdW complex

O	2.143286	-0.487668	-0.039738
O	1.235746	-0.884239	-0.975808
O	-0.554608	0.014942	1.383951
O	-0.870163	1.982402	-0.316535
C	-1.049126	-0.340649	0.327470
C	-1.364683	0.720647	-0.719446
C	2.092410	0.693079	0.381679
C	-1.483124	-1.757716	0.063032
H	-0.897536	0.438285	-1.664490
H	-2.456157	0.745209	-0.868432
H	1.341011	1.375151	-0.007574
H	2.830983	0.935829	1.136569
H	-2.549002	-1.848930	0.302989
H	-1.341394	-2.030751	-0.983055
H	-0.921059	-2.436391	0.702221
H	-0.813798	1.925934	0.650398

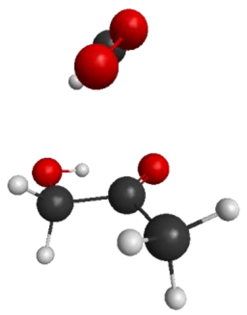

Temperature=	298.150000	Pressure=	1.000000
E(ZPE)=	0.121048	E(Thermal)=	0.131567
E(SCF)=	-455.548204	DE(MP2)=	-1.599088
DE(CBS)=	-0.158599	DE(MP34)=	-0.059012
DE(CCSD)=	-0.047841	DE(Int)=	0.050352
DE(Empirical)=	-0.076874		
CBS-QB3 (0 K)=	-457.318217	CBS-QB3 Energy=	-457.307698
CBS-QB3 Enthalpy=	-457.306753	CBS-QB3 Free Energy=	-457.354681

TS

O	-1.965292	0.185782	-0.267256
O	-0.934724	0.494106	-1.143599
O	0.035076	-0.001196	1.274782
O	1.303705	-1.795264	-0.207909
C	0.633288	0.486735	0.286488
C	1.547611	-0.435822	-0.516400
C	-1.739325	-0.878917	0.377235
C	0.887324	1.971865	0.203910
H	1.392757	-0.295462	-1.585872
H	2.588237	-0.154788	-0.282524
H	-1.030415	-1.601792	-0.006524
H	-2.443789	-1.083349	1.176063
H	1.725712	2.219921	0.865046
H	1.138565	2.283591	-0.810686
H	0.005939	2.513229	0.543677
H	1.139488	-1.811934	0.745272

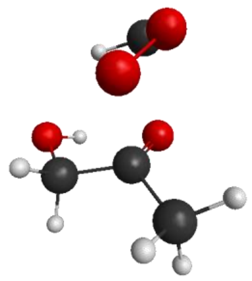

Temperature=	298.150000	Pressure=	1.000000
E(ZPE)=	0.122430	E(Thermal)=	0.131161
E(SCF)=	-455.534306	DE(MP2)=	-1.611561
DE(CBS)=	-0.159358	DE(MP34)=	-0.056076
DE(CCSD)=	-0.049160	DE(Int)=	0.050402
DE(Empirical)=	-0.076726		
CBS-QB3 (0 K)=	-457.314355	CBS-QB3 Energy=	-457.305624
CBS-QB3 Enthalpy=	-457.304679	CBS-QB3 Free Energy=	-457.347308

SOZ: (3-methyl-1,2,4-trioxolan-3-yl)methanol

O	1.795831	-0.233621	0.622883
O	0.482332	0.113424	1.152916
O	0.320788	-0.326622	-1.066185
O	-1.944576	-1.218976	0.132393
C	-0.221066	0.514613	-0.030452
C	-1.700356	0.166513	0.186895
C	1.396904	-1.032152	-0.464812
C	0.028507	1.974034	-0.365369
H	-2.001925	0.513970	1.177244
H	-2.287948	0.717553	-0.561153
H	1.062257	-2.018275	-0.123239
H	2.241623	-1.094327	-1.152860
H	-0.364668	2.207142	-1.356787
H	-0.457115	2.619260	0.371098
H	1.100883	2.169752	-0.354558
H	-1.552040	-1.526753	-0.693372

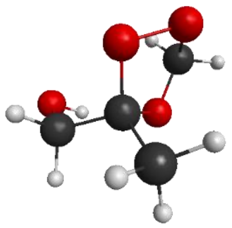

Temperature=	298.150000	Pressure=	1.000000
E(ZPE)=	0.126858	E(Thermal)=	0.134796
E(SCF)=	-455.615329	DE(MP2)=	-1.608945
DE(CBS)=	-0.159471	DE(MP34)=	-0.053912
DE(CCSD)=	-0.043045	DE(Int)=	0.049696
DE(Empirical)=	-0.077233		
CBS-QB3 (0 K)=	-457.381380	CBS-QB3 Energy=	-457.373443
CBS-QB3 Enthalpy=	-457.372499	CBS-QB3 Free Energy=	-457.413481

CH_2OO + hydroxyacetone (1,2-addition)

vdW complex

O	-3.059371	0.457564	0.301608
O	-1.897947	1.151948	0.171551
O	1.652390	1.213592	-0.527044
O	-0.126448	-0.856985	-0.621336
C	2.019601	0.170970	-0.041426
C	3.433348	-0.047885	0.460274
C	-3.103727	-0.767789	0.021441
C	1.056373	-1.004993	0.114040
H	3.923778	0.913247	0.609822
H	3.449117	-0.627540	1.387601
H	3.994269	-0.616654	-0.289199
H	-2.203323	-1.279553	-0.314373
H	-4.080100	-1.218209	0.155337
H	-0.490039	0.038326	-0.458954
H	0.871713	-1.115015	1.198194
H	1.552032	-1.925366	-0.212641

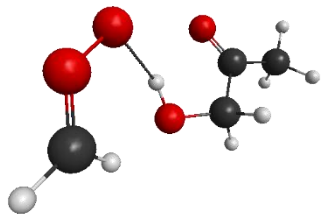

Temperature=	298.150000	Pressure=	1.000000
E(ZPE)=	0.120457	E(Thermal)=	0.131304
E(SCF)=	-455.543109	DE(MP2)=	-1.596985
DE(CBS)=	-0.158165	DE(MP34)=	-0.059262
DE(CCSD)=	-0.047140	DE(Int)=	0.050188
DE(Empirical)=	-0.076849		
CBS-QB3 (0 K)=	-457.310864	CBS-QB3 Energy=	-457.300016
CBS-QB3 Enthalpy=	-457.299072	CBS-QB3 Free Energy=	-457.349848

TS

O	-2.256617	-0.050361	0.797971
O	-1.819044	1.279121	0.398845
O	1.675224	1.229305	-0.557708
O	-0.490658	-0.344380	-0.904902
C	1.747418	0.132536	-0.064598
C	3.001583	-0.398454	0.599202
C	-2.310049	-0.748624	-0.265374
C	0.550073	-0.822619	-0.075354
H	3.683862	0.426584	0.798845
H	2.773095	-0.929312	1.527731
H	3.493847	-1.110469	-0.071978
H	-2.653683	-0.292533	-1.187198
H	-2.380955	-1.824549	-0.118372
H	-0.798261	0.589171	-0.509957
H	0.210664	-0.940720	0.965008
H	0.866052	-1.804680	-0.440978

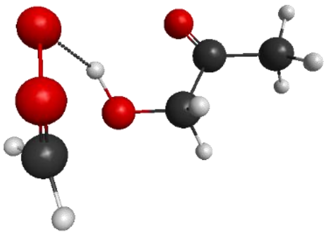

Temperature=	298.150000	Pressure=	1.000000
E(ZPE)=	0.120362	E(Thermal)=	0.129493
E(SCF)=	-455.525263	DE(MP2)=	-1.607980
DE(CBS)=	-0.158704	DE(MP34)=	-0.055787
DE(CCSD)=	-0.047109	DE(Int)=	0.050251
DE(Empirical)=	-0.076721		
CBS-QB3 (0 K)=	-457.300950	CBS-QB3 Energy=	-457.291819
CBS-QB3 Enthalpy=	-457.290875	CBS-QB3 Free Energy=	-457.336240

1-(hydroperoxymethoxy)propan-2-one

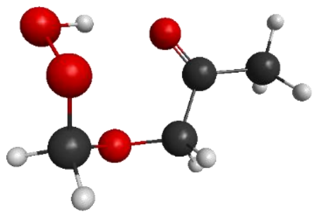

O	-1.680236	0.309073	0.810368
O	-1.876414	1.370424	-0.157744
O	0.904778	1.224538	-0.459753
O	-0.571217	-1.118230	-0.702442
C	1.416162	0.196369	-0.075064
C	2.859794	0.146200	0.375712
C	-1.697338	-0.905887	0.121026
C	0.670304	-1.138828	-0.043587
H	3.207493	1.153022	0.601617
H	2.990488	-0.503013	1.245643
H	3.473407	-0.265001	-0.433148
H	-2.557087	-0.968600	-0.546535
H	-1.748274	-1.653457	0.925139
H	-0.952015	1.495988	-0.452978
H	0.577640	-1.446353	1.009250
H	1.299531	-1.886149	-0.540950

Temperature=	298.150000	Pressure=	1.000000
E(ZPE)=	0.125057	E(Thermal)=	0.134014
E(SCF)=	-455.621664	DE(MP2)=	-1.600265
DE(CBS)=	-0.159284	DE(MP34)=	-0.055858
DE(CCSD)=	-0.041816	DE(Int)=	0.050044
DE(Empirical)=	-0.077147		
CBS-QB3 (0 K)=	-457.380933	CBS-QB3 Energy=	-457.371976
CBS-QB3 Enthalpy=	-457.371032	CBS-QB3 Free Energy=	-457.415165

CH_2OO + 4-hydroxy-2-butanone (cycloaddition A)

vdW complex

O	-2.398224	0.642831	0.211194
O	-1.542330	1.708457	0.389886
O	1.681862	-0.350823	-1.206438
O	-0.404643	-1.888225	-0.310406
C	-2.250679	-0.070101	-0.806984
C	1.587788	0.284513	-0.168570
C	0.867385	-0.272349	1.044760
C	0.395569	-1.715225	0.862940
C	2.115187	1.694026	-0.061652
H	-1.504959	0.203412	-1.543680
H	-2.928263	-0.908456	-0.899065
H	1.524732	-0.194074	1.919445
H	0.022775	0.406589	1.230869
H	-0.220019	-2.019355	1.711110
H	1.258995	-2.390570	0.817156
H	1.252269	2.367773	-0.022854
H	2.686124	1.842432	0.858488
H	2.725874	1.931760	-0.931690
H	0.197655	-1.662613	-1.036631

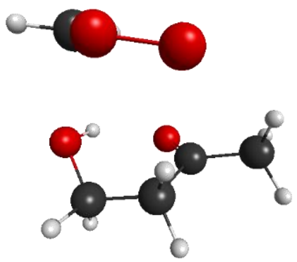

Temperature=	298.150000	Pressure=	1.000000
E(ZPE)=	0.149564	E(Thermal)=	0.161301
E(SCF)=	-494.599211	DE(MP2)=	-1.766668
DE(CBS)=	-0.174663	DE(MP34)=	-0.071478
DE(CCSD)=	-0.052237	DE(Int)=	0.056063
DE(Empirical)=	-0.085785		
CBS-QB3 (0 K)=	-496.544415	CBS-QB3 Energy=	-496.532679
CBS-QB3 Enthalpy=	-496.531734	CBS-QB3 Free Energy=	-496.583591

TS

O	-1.964303	-0.861070	0.682421
O	-1.740919	0.479037	0.951310
O	-0.136638	-0.339601	-1.038920
O	2.404055	-1.317115	-0.256276
C	-2.120683	-1.093635	-0.548752
C	0.080104	0.612464	-0.245052
C	1.058330	0.386699	0.894251
C	2.434649	-0.034931	0.342500
C	-0.210005	2.033162	-0.668274
H	-2.417286	-0.300009	-1.218532
H	-2.148887	-2.143556	-0.817820
H	1.150629	1.287111	1.506686
H	0.683148	-0.420205	1.525999
H	3.149272	-0.086470	1.167718
H	2.806385	0.725283	-0.363382
H	-0.536270	2.642692	0.174815
H	0.714184	2.470013	-1.066816
H	-0.963810	2.049238	-1.454671
H	1.610697	-1.336652	-0.810315

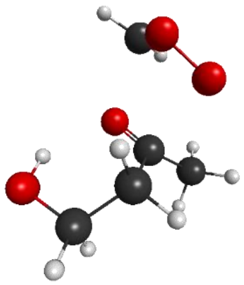

Temperature=	298.150000	Pressure=	1.000000
E(ZPE)=	0.150486	E(Thermal)=	0.160655
E(SCF)=	-494.582026	DE(MP2)=	-1.780977
DE(CBS)=	-0.175363	DE(MP34)=	-0.068655
DE(CCSD)=	-0.054448	DE(Int)=	0.056157
DE(Empirical)=	-0.085586		
CBS-QB3 (0 K)=	-496.540412	CBS-QB3 Energy=	-496.530244
CBS-QB3 Enthalpy=	-496.529300	CBS-QB3 Free Energy=	-496.576364

SOZ: 2-(3-methyl-1,2,4-trioxolan-3-yl)ethan-1-ol

O	-1.756964	-0.831702	0.810915
O	-1.523123	0.600262	0.688362
O	-0.367786	-0.489228	-0.925222
O	2.279963	-1.204217	-0.284389
C	-0.270524	0.635796	-0.013396
C	-1.513337	-1.223087	-0.520809
C	0.902002	0.425251	0.948903
C	2.242193	0.097021	0.278406
C	-0.248461	1.938858	-0.791082
H	-2.363835	-0.976562	-1.166587
H	-1.302393	-2.294256	-0.509183
H	1.005374	1.334218	1.551166
H	0.638801	-0.393677	1.622135
H	3.029802	0.126714	1.034736
H	2.493159	0.861511	-0.473194
H	-0.301365	2.786762	-0.105324
H	0.674408	2.014786	-1.367880
H	-1.097180	1.974768	-1.473757
H	1.495276	-1.278223	-0.841569

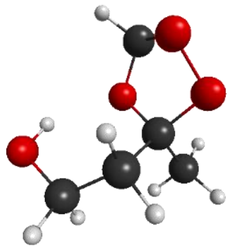

Temperature=	298.150000	Pressure=	1.000000
E(ZPE)=	0.155323	E(Thermal)=	0.164449
E(SCF)=	-494.662286	DE(MP2)=	-1.782567
DE(CBS)=	-0.175647	DE(MP34)=	-0.065727
DE(CCSD)=	-0.048171	DE(Int)=	0.055460
DE(Empirical)=	-0.086060		
CBS-QB3 (0 K)=	-496.609675	CBS-QB3 Energy=	-496.600549
CBS-QB3 Enthalpy=	-496.599605	CBS-QB3 Free Energy=	-496.643636

CH_2OO + 4-hydroxy-2-butanone (cycloaddition B)

vdW complex

O	2.403833	-0.459480	0.275280
O	1.522032	-0.259047	1.297004
O	-0.121833	0.184501	-1.268133
O	-2.401535	-1.292441	-0.501090
C	-0.387703	0.967792	-0.366147
C	-1.488523	0.680205	0.638901
C	2.114860	-1.293547	-0.614646
C	0.326166	2.292814	-0.242885
C	-1.874232	-0.797406	0.721682
H	-1.184484	1.060309	1.619252
H	-2.360898	1.271118	0.326246
H	1.190876	-1.854495	-0.551894
H	2.844047	-1.387260	-1.410108
H	-0.383107	3.104177	-0.056949
H	1.000684	2.232482	0.615306
H	0.900984	2.495280	-1.146207
H	-2.654660	-0.930893	1.474213
H	-0.995199	-1.368669	1.046647
H	-1.721624	-1.109459	-1.162425

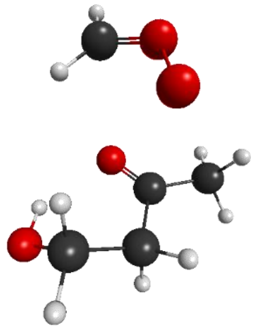

Temperature=	298.150000	Pressure=	1.000000
E(ZPE)=	0.149347	E(Thermal)=	0.161215
E(SCF)=	-494.597432	DE(MP2)=	-1.768378
DE(CBS)=	-0.174593	DE(MP34)=	-0.071607
DE(CCSD)=	-0.053132	DE(Int)=	0.056099
DE(Empirical)=	-0.085751		
CBS-QB3 (0 K)=	-496.545447	CBS-QB3 Energy=	-496.533579
CBS-QB3 Enthalpy=	-496.532635	CBS-QB3 Free Energy=	-496.584462

TS

O	2.086172	-0.719276	0.381411
O	1.192972	-0.101358	1.248303
O	0.123015	-0.131090	-1.172865
O	-2.500942	-0.886035	-0.522080
C	0.035937	0.791042	-0.317498
C	-1.225344	0.936922	0.519527
C	1.522461	-1.562474	-0.373942
C	0.918307	2.009956	-0.463093
C	-2.006211	-0.363876	0.703006
H	-0.964680	1.370958	1.488780
H	-1.861358	1.667512	0.000723
H	0.592407	-2.025863	-0.080013
H	2.146299	-1.943551	-1.175166
H	0.477341	2.655014	-1.232597
H	0.997161	2.574421	0.465279
H	1.911460	1.715931	-0.802157
H	-2.876193	-0.181496	1.338269
H	-1.366251	-1.088628	1.224538
H	-1.736821	-0.911657	-1.113809

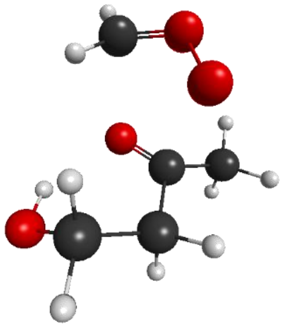

Temperature=	298.150000	Pressure=	1.000000
E(ZPE)=	0.150754	E(Thermal)=	0.160806
E(SCF)=	-494.582680	DE(MP2)=	-1.781946
DE(CBS)=	-0.175365	DE(MP34)=	-0.068652
DE(CCSD)=	-0.054641	DE(Int)=	0.056133
DE(Empirical)=	-0.085590		
CBS-QB3 (0 K)=	-496.541987	CBS-QB3 Energy=	-496.531935
CBS-QB3 Enthalpy=	-496.530991	CBS-QB3 Free Energy=	-496.577614

SOZ: 2-(3-methyl-1,2,4-trioxolan-3-yl)ethan-1-ol

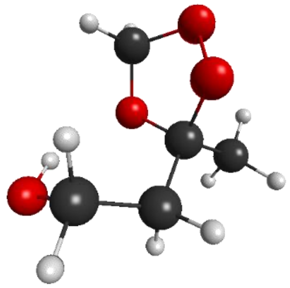

O	1.936522	-0.769075	0.602829
O	0.936010	0.146792	1.134386
O	0.303798	-0.527393	-0.929610
O	-2.494707	-0.748808	-0.531365
C	0.322503	0.639200	-0.065383
C	-1.092336	1.081765	0.313779
C	1.141613	-1.492245	-0.309031
C	1.159762	1.728888	-0.715472
C	-2.057138	-0.062967	0.632478
H	-0.998334	1.750880	1.175594
H	-1.502571	1.670138	-0.511876
H	0.531648	-2.244171	0.205586
H	1.817761	-1.943988	-1.037568
H	0.759666	1.964276	-1.703543
H	1.143495	2.632134	-0.101485
H	2.190159	1.389626	-0.822253
H	-2.954814	0.345667	1.101544
H	-1.591839	-0.746537	1.355518
H	-1.694587	-0.998002	-1.009662

Temperature=	298.150000	Pressure=	1.000000
E(ZPE)=	0.155341	E(Thermal)=	0.164426
E(SCF)=	-494.663002	DE(MP2)=	-1.782584
DE(CBS)=	-0.175532	DE(MP34)=	-0.065712
DE(CCSD)=	-0.048169	DE(Int)=	0.055429
DE(Empirical)=	-0.086060		
CBS-QB3 (0 K)=	-496.610290	CBS-QB3 Energy=	-496.601205
CBS-QB3 Enthalpy=	-496.600261	CBS-QB3 Free Energy=	-496.644171

CH_2OO + 4-hydroxy-2-butanone (1,2-addition)

vdWcomplex

O	-3.626287	0.211417	0.142005
O	-2.608533	0.677281	0.921367
O	3.412831	-0.672915	-0.502062
O	-0.367481	-0.098211	-0.489642
C	2.600594	0.068094	0.006430
C	2.518562	1.538631	-0.335633
C	-3.383882	-0.312054	-0.973912
C	0.361161	-1.074304	0.248331
C	1.568830	-0.476779	0.991177
H	1.517305	1.749708	-0.723157
H	2.652861	2.149304	0.563228
H	3.276576	1.794424	-1.075100
H	-2.353975	-0.408985	-1.316308
H	-4.265824	-0.643436	-1.509640
H	-1.006121	0.329521	0.117212
H	-0.291648	-1.579529	0.970651
H	0.720415	-1.819302	-0.465948
H	1.223419	0.298839	1.681533
H	2.051153	-1.272645	1.565824

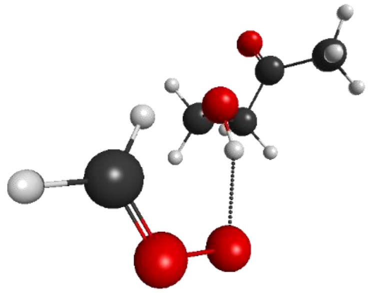

Temperature=	298.150000	Pressure=	1.000000
E(ZPE)=	0.149596	E(Thermal)=	0.161492
E(SCF)=	-494.593459	DE(MP2)=	-1.767968
DE(CBS)=	-0.174184	DE(MP34)=	-0.071244
DE(CCSD)=	-0.051914	DE(Int)=	0.055871
DE(Empirical)=	-0.085735		
CBS-QB3 (0 K)=	-496.539037	CBS-QB3 Energy=	-496.527140
CBS-QB3 Enthalpy=	-496.526196	CBS-QB3 Free Energy=	-496.579949

TS

O	-2.911251	0.266088	0.352560
O	-2.245349	1.440733	-0.185661
O	2.517797	-1.194677	-0.384012
O	-0.736650	-0.520808	-0.549877
C	2.269298	-0.075752	0.000193
C	3.133847	1.113936	-0.356212
C	-2.713905	-0.687807	-0.467222
C	-0.078878	-0.778609	0.703402
C	1.066771	0.202037	0.905918
H	2.573058	1.779918	-1.020985
H	3.386748	1.695491	0.535716
H	4.040791	0.780037	-0.858305
H	-2.699941	-0.484672	-1.532064
H	-2.943294	-1.683189	-0.093283
H	-1.055411	0.466800	-0.521195
H	-0.798603	-0.683215	1.523618
H	0.301065	-1.798934	0.656486
H	0.706926	1.229365	0.789580
H	1.429489	0.124888	1.939886

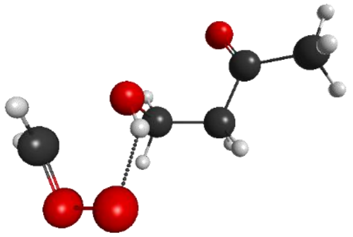

Temperature=	298.150000	Pressure=	1.000000
E(ZPE)=	0.149574	E(Thermal)=	0.159937
E(SCF)=	-494.578617	DE(MP2)=	-1.778608
DE(CBS)=	-0.174800	DE(MP34)=	-0.068073
DE(CCSD)=	-0.051942	DE(Int)=	0.056004
DE(Empirical)=	-0.085598		
CBS-QB3 (0 K)=	-496.532060	CBS-QB3 Energy=	-496.521698
CBS-QB3 Enthalpy=	-496.520754	CBS-QB3 Free Energy=	-496.570101

4-(hydroperoxymethoxy)butan-2-one

O	2.906055	0.338444	0.297909
O	3.469808	-0.740006	-0.490995
O	-2.228364	1.323688	0.067957
O	0.684591	-0.060522	-0.333211
C	-2.317530	0.119525	0.009237
C	-3.433983	-0.570781	-0.744989
C	1.770298	0.819165	-0.387818
C	0.031726	-0.126820	0.940782
C	-1.313752	-0.790790	0.721234
H	-3.018549	-1.053466	-1.636234
H	-3.890535	-1.358674	-0.138250
H	-4.186214	0.156754	-1.046714
H	2.003826	0.961630	-1.443368
H	1.552185	1.769775	0.114906
H	2.884868	-1.475058	-0.254820
H	0.640323	-0.695415	1.651906
H	-0.116745	0.885329	1.332738
H	-1.188016	-1.725846	0.167362
H	-1.758417	-1.059652	1.688517

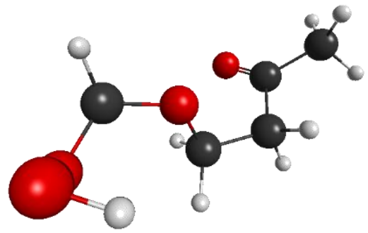

Temperature=	298.150000	Pressure=	1.000000
E(ZPE)=	0.152340	E(Thermal)=	0.163274
E(SCF)=	-494.668349	DE(MP2)=	-1.769346
DE(CBS)=	-0.175341	DE(MP34)=	-0.068284
DE(CCSD)=	-0.046357	DE(Int)=	0.055820
DE(Empirical)=	-0.086018		
CBS-QB3 (0 K)=	-496.605537	CBS-QB3 Energy=	-496.594602
CBS-QB3 Enthalpy=	-496.593658	CBS-QB3 Free Energy=	-496.644123

References

(1) Johnson, T. J.; Profeta, L. T. M.; Sams, R. L.; Griffith, D. W. T.; Yokelson, R. L. An Infrared Spectral Database for Detection of Gases Emitted by Biomass Burning. *Vib. Spectrosc.* **2010**, *53* (1), 97–102. https://doi.org/10.1016/j.vibspec.2010.02.010.

(2) Petitjean, M.; Reyès-Pérez, E.; Pérez, D.; Mirabel, Ph.; Le Calvé, S. Vapor Pressure Measurements of Hydroxyacetaldehyde and Hydroxyacetone in the Temperature Range (273 to 356) K. *J. Chem. Eng. Data* **2010**, *55* (2), 852–855. https://doi.org/10.1021/je9004905.

(3) El Dib, G.; Sleiman, C.; Canosa, A.; Travers, D.; Courbe, J.; Sawaya, T.; Mokbel, I.; Chakir, A. First Experimental Determination of the Absolute Gas-Phase Rate Coefficient for the Reaction of OH with 4-Hydroxy-2-Butanone (4H2B) at 294 K by Vapor Pressure Measurements of 4H2B. *J. Phys. Chem. A* **2013**, *117* (1), 117–125. https://doi.org/10.1021/jp3074909.

(4) Keller-Rudek, H.; Moortgat, G. K.; Sander, R.; Sörensen, R. The MPI-Mainz UV/VIS Spectral Atlas of Gaseous Molecules of Atmospheric Interest. *Earth Syst. Sci. Data* **2013**, *5*, 365–373. https://doi.org/10.5194/essd-5-365-2013.

(5) *J. B. Burkholder, S. P. Sander, J. Abbatt, J. R. Barker, C. Cappa, J. D. Crounse, T. S. Dibble, R. E. Huie, C. E. Kolb, M. J. Kurylo, V. L. Orkin, C. J. Percival, D. M. Wilmouth, and P. H. Wine "Chemical Kinetics and Photochemical Data for Use in Atmospheric Studies, Evaluation No. 19," JPL Publication 19-5, Jet Propulsion Laboratory, Pasadena, 2019 Http://Jpldataeval.Jpl.Nasa.Gov*; 19; 2019.

(6) *IUPAC – Task Group on Atmospheric Chemical Kinetic Data Evaluation*. https://iupac.aeris-data.fr/ (accessed 2023-06-23).

(7) Messaadia, L.; El Dib, G.; Ferhati, A.; Roth, E.; Chakir, A. Gas Phase UV Absorption Cross-Sections for a Series of Hydroxycarbonyls. *Chem. Phys. Lett.* **2012**, *529*, 16–22. https://doi.org/10.1016/j.cplett.2012.01.044.

(8) Cornwell, Z. A.; Enders, J. J.; Harrison, A. W.; Murray, C. Temperature-Dependent Kinetics of the Reactions of CH_2OO with Acetone, Biacetyl, and Acetylacetone. *Int. J. Chem. Kinet.* **2023**, *55* (3), 154–166. https://doi.org/10.1002/kin.21625.

(9) Luo, P.-L.; Chen, I.-Y.; Khan, M. A. H.; Shallcross, D. E. Direct Gas-Phase Formation of Formic Acid through Reaction of Criegee Intermediates with Formaldehyde. *Commun. Chem.* **2023**, *6* (1), 1–10. https://doi.org/10.1038/s42004-023-00933-2.

(10) Stone, D.; Blitz, M.; Daubney, L.; Howes, N. U. M.; Seakins, P. Kinetics of CH_2OO Reactions with SO_2, NO_2, NO, H_2O and CH_3CHO as a Function of Pressure. *Phys. Chem. Chem. Phys.* **2014**, *16* (3), 1139–1149. https://doi.org/10.1039/C3CP54391A.

(11) Taatjes, C. A.; Welz, O.; Eskola, A. J.; Savee, J. D.; Osborn, D. L.; Lee, E. P. F.; Dyke, J. M.; Mok, D. W. K.; Shallcross, D. E.; Percival, C. J. Direct Measurement of Criegee Intermediate (CH_2OO) Reactions with Acetone, Acetaldehyde, and Hexafluoroacetone. *Phys. Chem. Chem. Phys.* **2012**, *14* (30), 10391–10400. https://doi.org/10.1039/C2CP40294G.

(12) Elsamra, R. M. I.; Jalan, A.; Buras, Z. J.; Middaugh, J. E.; Green, W. H. Temperature- and Pressure-Dependent Kinetics of CH_2OO + CH_3COCH_3 and CH_2OO + CH_3CHO: Direct Measurements and Theoretical Analysis. *Int. J. Chem. Kinet.* **2016**, *48* (8), 474–488. https://doi.org/10.1002/kin.21007.

(13) Berndt, T.; Kaethner, R.; Voigtländer, J.; Stratmann, F.; Pfeifle, M.; Reichle, P.; Sipilä, M.; Kulmala, M.; Olzmann, M. Kinetics of the Unimolecular Reaction of CH_2OO and the Bimolecular Reactions with the Water Monomer, Acetaldehyde and Acetone under Atmospheric Conditions. *Phys. Chem. Chem. Phys.* **2015**, *17* (30), 19862–19873. https://doi.org/10.1039/C5CP02224J.

(14) Chhantyal-Pun, R.; Khan, M. A. H.; Martin, R.; Zachhuber, N.; Buras, Z. J.; Percival, C. J.; Shallcross, D. E.; Orr-Ewing, A. J. Direct Kinetic and Atmospheric Modeling Studies of Criegee Intermediate Reactions with Acetone. *ACS Earth Space Chem.* **2019**, *3* (10), 2363–2371. https://doi.org/10.1021/acsearthspacechem.9b00213.

(15) Debnath, A.; Rajakumar, B. Investigation of Kinetics and Mechanistic Insights of the Reaction of Criegee Intermediate (CH_2OO) with Methyl-Ethyl Ketone (MEK) under Tropospherically Relevant Conditions. *Chemosphere* **2023**, *312*, 137217. https://doi.org/10.1016/j.chemosphere.2022.137217.
(16) Liu, Y.; Bayes, K. D.; Sander, S. P. Measuring Rate Constants for Reactions of the Simplest Criegee Intermediate (CH_2OO) by Monitoring the OH Radical. *J. Phys. Chem. A* **2014**, *118* (4), 741–747. https://doi.org/10.1021/jp407058b.
(17) Cornwell, Z. A.; Harrison, A. W.; Murray, C. Kinetics of the Reactions of CH_2OO with Acetone, α-Diketones, and β-Diketones. *J. Phys. Chem. A* **2021**, *125* (39), 8557–8571. https://doi.org/10.1021/acs.jpca.1c05280.
(18) Eskola, A. J.; Döntgen, M.; Rotavera, B.; Caravan, R. L.; Welz, O.; Savee, J. D.; Osborn, D. L.; Shallcross, D. E.; Percival, C. J.; Taatjes, C. A. Direct Kinetics Study of CH_2OO + Methyl Vinyl Ketone and CH_2OO + Methacrolein Reactions and an Upper Limit Determination for CH_2OO + CO Reaction. *Phys. Chem. Chem. Phys.* **2018**, *20* (29), 19373–19381. https://doi.org/10.1039/C8CP03606C.
(19) Zhou, X.; Chen, Y.; Liu, Y.; Li, X.; Dong, W.; Yang, X. Kinetics of CH_2OO and Syn-CH_3CHOO Reaction with Acrolein. *Phys. Chem. Chem. Phys.* **2021**, *23* (23), 13276–13283. https://doi.org/10.1039/D1CP00492A.
